# Artificial intelligence–based diagnosis of diabetic kidney disease using urinary VOC biosensor data

**DOI:** 10.1186/s12882-025-04608-z

**Published:** 2025-11-26

**Authors:** Chatchai Kreepala, Watcharapong Anakkamatee, Anawin Pechbooranin

**Affiliations:** 1 Nephrology Unit, School of Internal Medicine, Institute of Medicine, Suranaree University of Technology, 111 University Avenue, Suranaree Subdistrict, Mueang Nakhon Ratchasima District, Nakhon Ratchasima, 30000 Thailand; 2 Department of Mathematics, Faculty of Science, Naresuan University, Phitsanulok, Thailand; 3 School of Mechatronics Engineering, Institute of Engineering, Suranaree University of Technology, Nakhon Ratchasima, Thailand

**Keywords:** VOCs, Biosensor, Diabetic kidney disease, Artificial intelligence, Urine analysis

## Abstract

**Background:**

Diabetic kidney disease (DKD) remains a leading cause of chronic kidney disease worldwide. However, current diagnostic methods rely on indirect biomarkers or invasive renal biopsy. This study aimed to evaluate the feasibility of urinary volatile organic compound (VOC) profiling, combined with machine learning, for non-invasive classification of DKD.

**Methods:**

Urine samples were collected from 127 participants divided into four diagnostic groups: healthy controls, patients with type 2 diabetes without nephropathy, biopsy-confirmed DKD, and patients with primary nephrotic syndromes. Samples were analyzed using a chemiresistive VOC biosensor. A total of 15,240 signal-derived features were extracted based on sensor response dynamics. Synthetic Minority Over-sampling Technique (SMOTE) was applied to balance class sizes. Four machine learning classifiers—Random Forest, Support Vector Machine, k-Nearest Neighbors, and Naïve Bayes—were trained and validated using stratified data. Performance was assessed using accuracy, precision, recall, F1-score, and area under the receiver operating characteristic curve (AUC).

**Results:**

Random Forest achieved the best test performance, with 86% accuracy, 0.91 precision, 0.86 recall, F1-score of 0.86, and an AUC of 0.95. K-fold cross-validation confirmed the model’s robustness and generalizability. Random Forest consistently outperformed other models in distinguishing DKD from both diabetic and non-diabetic glomerular diseases, demonstrating its strong discriminative capability.

**Conclusions:**

Urinary VOC-based machine learning models provide proof-of-concept evidence for non-invasive DKD diagnosis. Random Forest, in particular, shows potential as a triage tool to differentiate DKD from other glomerular conditions, which may in the future help reduce reliance on biopsy and support earlier identification in nephrology practice.

**Supplementary Information:**

The online version contains supplementary material available at 10.1186/s12882-025-04608-z.

## Introduction

### Background

Diabetic kidney disease (DKD) is a leading cause of chronic kidney disease (CKD) worldwide, affecting 20–40% of individuals with type 2 diabetes mellitus (DM) [1, 2]. Diagnosis in clinical practice relies mainly on albuminuria and estimated glomerular filtration rate (eGFR) [3]. However, these markers lack specificity for distinguishing DKD from other glomerular diseases such as glomerulonephritis (GN), which often share overlapping clinical and laboratory features. Although renal biopsy remains the diagnostic gold standard, it is invasive, costly, and associated with risks [4, 5]. In many cases, physicians assume DKD as the default etiology, which may lead to missed opportunities for diagnosing treatable or reversible conditions [6–8].

In search of non-invasive alternatives, urinary volatile organic compounds (VOCs) have emerged as promising biomarkers. VOCs reflect metabolic and inflammatory processes and can serve as biochemical fingerprints of disease states [9–11]. Our group previously developed a semiconductor-based biosensor to detect urinary VOCs and demonstrated its ability to differentiate DKD from other nephropathies [9]. Nonetheless, the interpretation of multidimensional VOC signal patterns remains challenging without advanced computational tools. This highlights the need for AI/ML approaches to extract clinically meaningful patterns from VOC biosensor data.

### Related work

In recent years, artificial intelligence (AI) and machine learning (ML) have transformed medical diagnostics, including imaging-based detection of diabetic retinopathy and nephropathy [12–14]. Similarly, VOC-based approaches have been explored in lung cancer, liver disease, and uremia [11]. However, most prior studies applied gas chromatography–mass spectrometry (GC–MS) or proteomic platforms rather than practical biosensors. Furthermore, many reports relied on traditional statistical comparisons rather than building predictive AI models.

Several recent works (2022–2025) have applied AI to kidney disease diagnostics, but the majority focused on imaging or conventional biomarkers rather than VOC sensing [9, 10, 15–17]. A summary of these studies—covering population, methods, outcomes, and limitations—is provided in Table 1. Only a few reports have attempted AI-based modeling in nephrology, and these primarily focused on imaging or clinical biomarkers rather than VOCs. This underscores the novelty of integrating AI with biosensor-derived VOC signals for DKD.

**Table 1:** Summary of related studies on DKD diagnostics (2022–2025)

Study (Author, Year)	Population (*n*)	Methodology	Key Findings	Limitations
Kreepala et al., 2024 (Sci Rep) [10]	64 (renal cell carcinoma, bladder cancer, prostate cancer, controls)	Semiconductor VOC biosensor; statistical signal analysis	Identified urinary VOC patterns distinguishing urinary cancers from controls	No AI; focused on cancer, not DKD
Kreepala et al., 2025 (Sci Rep) [9]	135 (biopsy-proven DKD, DM, GN, controls)	Semiconductor VOC biosensor; feature-based statistical modeling	Demonstrated VOC biosensor ability to differentiate DKD from other nephropathies	No ML; limited handling of class imbalance
Wu et al., 2024 (BMC Nephrol) [15]	64 (biopsy patients, grouped by IFTA stage)	Urinary VOCs by GC–MS; linear discriminant analysis (LDA) with cross-validation	Identified urinary VOCs associated with CKD progression and fibrosis	Small sample; not DKD-specific; GC–MS not scalable
Ewen et al., 2024 (Molecules) [16]	30 (CKD patients across stages + controls)	Breath VOCs by TD–GC/MS; principal component analysis (PCA) and LDA	VOCs discriminated CKD stages with ~ 87–100% accuracy	Very small cohort; no DKD subgroup; expensive instrumentation
Lee et al., 2025 (J Clin Med) [17]	5,120 T2DM patients (Asian population)	Machine learning (XGBoost, RF, LR) using clinical/EHR data	Predicted DKD with AUC ~ 0.81, externally validated	Not VOC-based; relies on clinical data

Abbreviations: DKD, diabetic kidney disease; DM, diabetes mellitus; GN, glomerulonephritis; RCC, renal cell carcinoma; VOC, volatile organic compounds; GC–MS, gas chromatography–mass spectrometry; TD–GC/MS, thermal desorption gas chromatography–mass spectrometry; PCA, principal component analysis; LDA, linear discriminant analysis; AI, artificial intelligence; ML, machine learning

### Research gap and problems

Several recent studies have explored VOCs for kidney disease diagnostics, but most have relied on expensive, laboratory-based platforms such as GC–MS, limiting scalability in clinical practice [15, 16]. Moreover, prior VOC research has largely addressed CKD in general, without focusing specifically on DKD as a distinct entity [15, 16]. In parallel, AI applications for DKD prediction have emerged, yet these predominantly use clinical and electronic health record data, which lack disease-specific molecular signatures [17], albeit in Asian populations similar to ours.

To address these gaps, the present study builds directly on our previous biosensor investigations [9, 10], which demonstrated the feasibility of semiconductor-based VOC detection but did not apply AI-driven modeling. Here, we integrate machine learning with a cost-effective biosensor platform to tackle the critical diagnostic challenge of differentiating DKD from other glomerular diseases.

The key contributions of this study are threefold. First, it represents the novel integration of a semiconductor-based VOC biosensor with machine learning for DKD diagnosis, an approach not previously reported. Second, it achieves clinical specificity by differentiating DKD from biopsy-proven glomerular diseases, thereby addressing a major diagnostic challenge in nephrology. Third, it introduces a robust technical framework that combines feature selection, data balancing, and comparative evaluation of multiple classifiers, demonstrating that Random Forest provides the most reliable performance for this application.

## Materials and methods

### Clinical data collection and study design

This study was a secondary data analysis based on a previously published cohort that investigated the diagnostic utility of urinary VOCs for detecting DKD using a semiconductor-based biosensor platform [9]. The original study enrolled participants from Suranaree University of Technology Hospital between March 2024 and July 2024, aiming to distinguish DKD from other glomerular diseases using urinary VOC profiles measured by a chemiresistive sensor array.

The original cohort included patients aged over 20 years who were able to provide informed consent. Patients were classified into four groups for comparative analysis: (1) healthy controls, (2) individuals with type 2 diabetes but without evidence of DKD (DM without DKD), (3) biopsy-confirmed DKD cases, and (4) patients with biopsy-confirmed glomerular diseases unrelated to diabetes, such as primary GN or nephrotic syndrome (NS). Clinical inclusion criteria and laboratory parameters were described in detail in the original study protocol. Notably, subjects with ambiguous pathology findings, mixed etiologies, or advanced kidney failure (eGFR < 15 mL/min/1.73 m²) were excluded.

Diagnostic confirmation in the DKD group was based on kidney biopsy demonstrating Class II diabetic glomerulosclerosis, consistent with the 2010 pathological classification of diabetic nephropathy. Control groups without biopsy confirmation were required to demonstrate persistently normal urine albumin levels (< 30 mg/g creatinine) and eGFR > 60 mL/min/1.73 m² on at least two occasions spaced four weeks apart. Patients with intermediate (30–300 mg/g) albuminuria or uncertain clinical profiles were excluded to ensure diagnostic clarity.

All biospecimens and associated clinical data used in this analysis were obtained under prior ethical approval (Suranaree University of Technology Ethics Committee, EC-67-0001). The current study protocol was additionally reviewed and approved as a secondary data analysis without new patient recruitment or intervention.

### Gas sensors

A previously developed biosensor system was used to measure urinary VOCs via a chemiresistive sensor array composed of five commercially available metal oxide semiconductor sensors (Figaro Engineering Inc., Japan). The system captured changes in electrical resistance as VOCs interacted with each sensor under varying thermal conditions. Each sensor produced signal profiles across **six heating cycles** with five voltage levels (2000–5000 mV) [10].

From each sensor, characteristic features were extracted, including the minimum resistance value (Min), resistance drop (Gap), time to minimum (Time), and response slope (Slope) for each voltage cycle. A total of ~ 1200 features per subject were generated and used as input for machine learning analysis [10].

### Artificial intelligence-based classification and evaluation of DKD

Each urine sample was analyzed using five different gas sensors. Signal responses from each sensor were processed across six heating cycles, with four signal-derived features extracted per cycle. This yielded a total of 15,240 features across the dataset (127 subjects × 5 sensors × 4 features × 6 cycles). For machine learning development, secondary analysis was performed using these 127 samples with complete VOC signal data [9]. Data preprocessing involved transforming raw sensor resistance values into structured parameters across multiple sensors and heating cycles. After verifying data completeness, correlation analysis was applied to identify highly informative features for model development.

Because the dataset showed *moderate class imbalance*, particularly in the non-diabetic nephrotic syndrome (NS) group, we applied the Synthetic Minority Oversampling Technique (SMOTE) *only within the training folds after stratified 75:25 splitting*. *This design prevented synthetic samples from contaminating the independent test set*,* thereby avoiding data leakage and preserving the validity of performance estimates*. *The 75:25 split ratio was selected as a compromise between maximizing training data for model learning and retaining a sufficiently large test set for unbiased evaluation—an important consideration in studies with moderate sample size (n = 127). Stratified sampling was used to maintain proportional representation of diagnostic groups in both sets.*

Machine learning classifiers—including Random Forest (RF), Support Vector Machine (SVM), K-Nearest Neighbors (KNN), and Naïve Bayes (NB)—were implemented using Python scikit-learn. Feature scaling was applied using Standard score normalization (StandardScaler). Hyperparameters were optimized using GridSearchCV with 5-fold stratified cross-validation. This ensured that tuning and internal performance estimation were restricted to the training set, while the final model evaluation was conducted exclusively on the untouched test set.

Among the evaluated models, RF consistently demonstrated the highest discriminative performance, achieving a training accuracy of 89%, mean cross-validated accuracy of 82%, and test accuracy of 86%. The AUC-ROC for DKD classification reached 0.98, indicating robust separation between diagnostic groups under internal validation. While these results are promising, they remain preliminary and require confirmation in external cohorts.

**Fig. 1:**
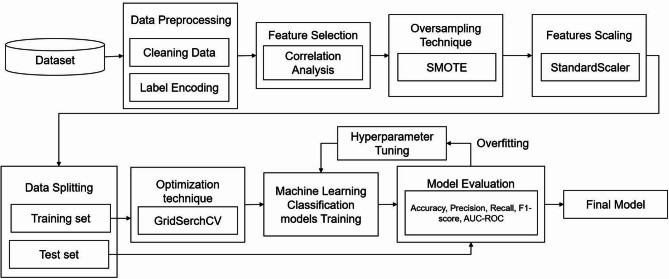
Workflow of the AI-based classification pipeline for DKD. Data preprocessing included label encoding and quality filtering. Feature selection was conducted using correlation analysis, retaining features with absolute correlation coefficients ≥ 0.80. To address class imbalance, SMOTE oversampling was applied, followed by feature scaling using the StandardScaler method. The dataset was split into training and test sets using stratified sampling. Hyperparameter tuning was performed using GridSearchCV. Multiple machine learning classifiers were trained and evaluated based on accuracy, precision, recall, F1-score, and AUC-ROC to select the final model. Abbreviations: AUC-ROC: Area under the receiver operating characteristic curve, F1-score: Harmonic mean of precision and recall, GridSearchCV: Grid search with cross-validation, SMOTE: Synthetic Minority Over-sampling Technique StandardScaler: Standard score normalization method used for feature scaling

This integrated workflow—from feature extraction and preprocessing to classifier comparison—demonstrates the feasibility of combining urinary VOC biosensor data with machine learning for the non-invasive diagnosis of DKD. The AI classification pipeline is illustrated in Fig. 1.

### Statistical analysis

Data preprocessing and exploratory analyses were performed using SPSS version 18.0 [18] and R version 2.9.1 [19], which were applied primarily for descriptive statistics and correlation testing. Continuous variables were summarized as mean with standard deviation (SD), and categorical variables as percentages. Group comparisons for categorical variables were conducted using the Pearson chi-square test. Spearman’s rank correlation analysis was used to evaluate monotonic relationships between signal-derived features and diagnostic categories.

All machine learning analyses—including feature scaling, model training, hyperparameter tuning, and performance evaluation—were conducted exclusively in Python version 3.10 [20] using the scikit-learn library. Model performance was assessed using accuracy, precision, recall, F1-score, and the area under the receiver operating characteristic curve (AUC-ROC). To mitigate class imbalance, SMOTE was applied only within training folds. Stratified 5-fold cross-validation was used during hyperparameter optimization, while independent test sets were reserved for final evaluation. In addition, to strengthen robustness, external cross-validation with bagging ensembles was performed, providing complementary estimates of model stability across folds. Statistical significance was defined as *p* ≤ 0.05.

## Results

### Dataset description and cohort overview

This study included 127 participants whose urine samples had previously been analyzed using a VOC biosensor platform. Participants were divided into four diagnostic groups: (1) healthy controls (*n* = 17, 13.4%), (2) individuals with type 2 diabetes mellitus but no kidney involvement (DM, *n* = 52, 40.9%), (3) biopsy-confirmed diabetic kidney disease (DKD, *n* = 50, 39.4%), and (4) non-diabetic nephrotic syndrome (NS, *n* = 8, 6.3%), including lupus nephritis, membranous nephropathy, IgA nephropathy, focal segmental glomerulosclerosis, and minimal change disease. Group classification followed the criteria outlined in the Methods.

Demographic and clinical characteristics, including age, fasting blood glucose, HbA1C, and urine protein levels, differed across the groups. Patients with NS had significantly higher levels of proteinuria compared to all other groups (*p* < 0.001). Among diabetic patients, HbA1C levels were elevated in both DM groups, but no statistically significant difference in glycemic control was observed between those with and without DKD. The healthy control group showed normal values in all measured parameters.

### Feature extraction and dimensionality

Urine samples from all 127 participants were analyzed using five distinct metal oxide semiconductor sensors. Signal responses from each sensor were processed across six heating cycles, with four signal-derived features extracted per cycle—namely minimum resistance, slope, signal gap, and response time. This yielded a total of 15,240 structured features across the dataset (127 samples × 5 sensors × 4 features × 6 cycles), which served as input for the AI-based classification model.

To prepare the dataset for machine learning, categorical variables were transformed into numerical representations using label encoding, ensuring compatibility with standard ML algorithms.

### Feature selection by correlation analysis

From the full set of 15,240 signal-derived features, feature selection was performed to reduce dimensionality and identify the most informative parameters for classification. Given the non-parametric distribution of the VOC signal data, Spearman’s rank correlation analysis was applied to assess the strength of association between each feature and the diagnostic group labels.

To ensure both statistical and practical relevance, only features with an absolute correlation coefficient (|r|) ≥ 0.80 and *p* < 0.01 were retained. This threshold was chosen to isolate features with consistently strong monotonic relationships, regardless of direction (positive or negative).

As a result, correlation analysis identified 13 high-correlation features from the complete dataset of 15,240 sensor-derived parameters (127 samples × 5 sensors × 4 features × 6 cycles). These features were subsequently used for model training.

A summary of the selected features and their correlation coefficients is provided in Table 2.

**Table 2:** Top 13 VOC-Derived features with strongest correlation to diagnostic group classification

Features	Correlation
Sensor 1 min Cycle 1	-0.80
Sensor 2 min Cycle 1	-0.84
Sensor 3 min Cycle 2	-0.86
Sensor 3 time Cycle 2	-0.83
Sensor 3 min Cycle 3	-0.84
Sensor 3 min Cycle 4	-0.81
Sensor 3 time Cycle 6	-0.81
Sensor 4 min Cycle 1	-0.82
Sensor 5 min Cycle 1	-0.82
Sensor 5 min Cycle 2	-0.82
Sensor 3 gap Cycle 2	0.85
Sensor 3 gap Cycle 3	0.84
Sensor 3 gap Cycle 4	0.82

Signal-derived parameters were selected based on Spearman’s rank correlation analysis. Features with ***absolute correlation coefficients (|r|) ≥ 0.80 and p < 0.001 were retained for model training.*** Both positively and negatively correlated features were included to capture diverse discriminatory signals

Abbreviation: VOC, Volatile organic compounds

### Handling imbalanced data

The original dataset demonstrated a notable class imbalance, with the NS and normal groups being underrepresented compared to the DKD and DM groups. Such imbalance can introduce bias into machine learning models, often leading to suboptimal performance in minority classes and overfitting to majority patterns.

To mitigate this issue, SMOTE was applied. It generates synthetic samples by interpolating between existing instances within the minority class, rather than duplicating them. This enhances class representation and allows the model to learn more balanced decision boundaries.

After applying SMOTE, the dataset was augmented to include 50 samples each for the DKD, NS, and normal groups, while the DM group retained 52 original samples—resulting in a total of 202 samples (see Supplementary Data). This balanced class distribution improved model robustness and ensured equitable performance across diagnostic categories.

### Model development and overfitting prevention

1. **Feature Scaling**

To ensure that all features contributed equally to the learning process—particularly for distance-sensitive algorithms such as KNN and SVM—feature scaling was applied using the StandardScaler method. This technique standardizes each feature by subtracting the mean and scaling to unit variance according to the formula: z = (x − µ) / σ, where z is the standardized value, x is the original feature value, µ is the mean, and σ is the standard deviation of the feature within the training set.

2. **Data Splitting**

The dataset was divided into a training set and a test set using a 75:25 split. Stratified sampling was employed to preserve the proportional representation of diagnostic groups in both sets. The training set was used for model fitting and internal validation, while the test set was held out for final model evaluation.

3. **Hyperparameter Optimization**

**Fig. 2:**
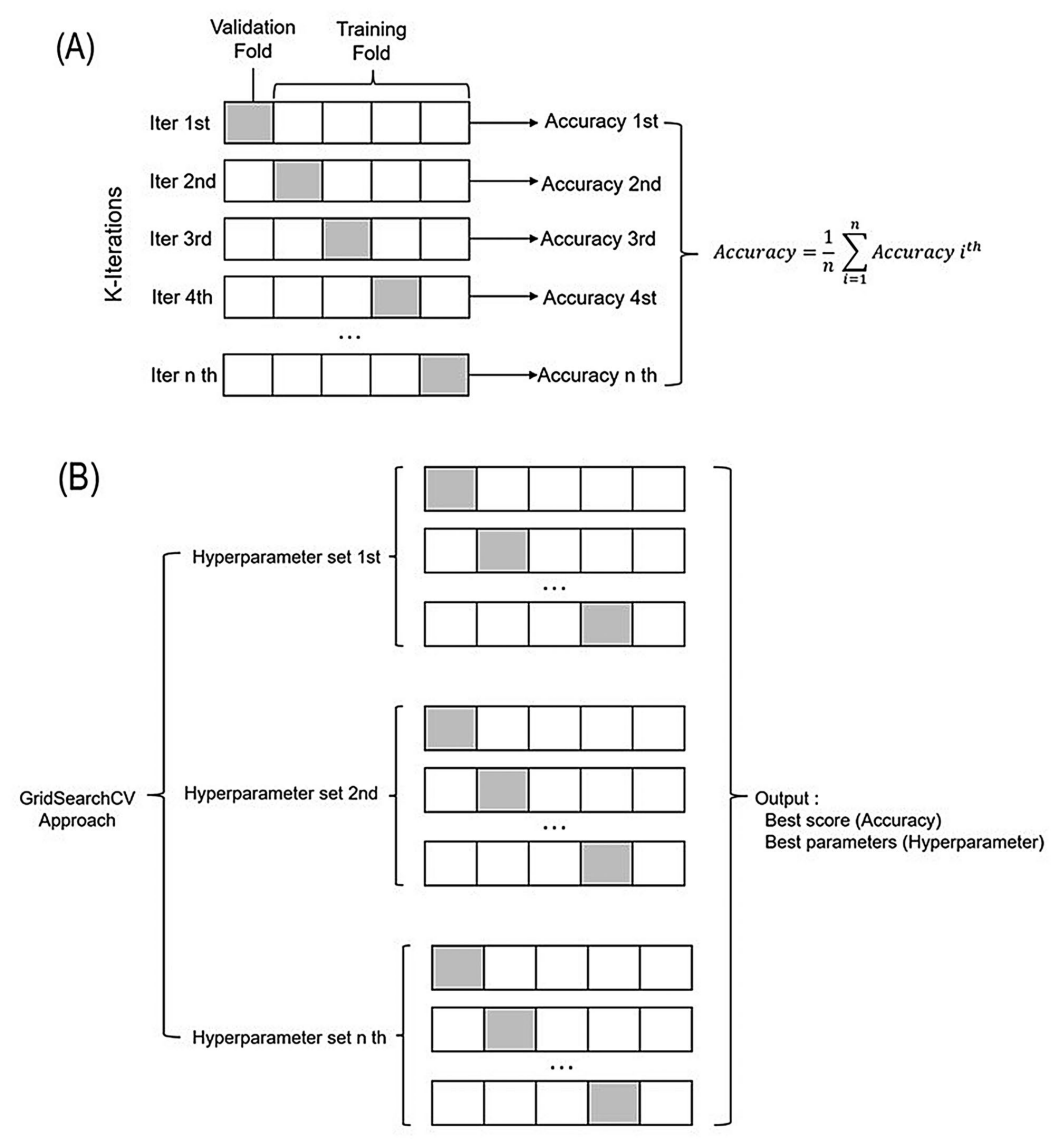
Hyperparameter tuning and cross-validation workflow. **(A)** GridSearchCV was used to identify optimal hyperparameters by systematically evaluating different parameter combinations using cross-validation within the training set. **(B)** A 5-fold cross-validation strategy was applied, in which the training data were split into five equal partitions. Each fold was used once for validation while the remaining four were used for training. The average performance across folds guided hyperparameter selection and minimized the risk of overfitting. Abbreviations: CV: Cross-validation; GridSearchCV: Grid search with cross-validation; KNN: K-Nearest Neighbors; SVM: Support Vector Machine

Hyperparameter tuning was conducted using GridSearchCV, which systematically explores combinations of model parameters to identify the configuration yielding the best performance. A 5-fold cross-validation strategy was incorporated within the training set: in each iteration, four folds were used for training and one for validation, cycling through all partitions. This approach enhanced model robustness while minimizing overfitting and variance (Fig. 2).

### Model evaluation

Following data preprocessing, feature selection, SMOTE-based class balancing, and hyperparameter optimization using 5-fold cross-validation, four machine learning models—RF, SVM, KNN, and NB—were trained and evaluated. The assessment included training accuracy, internal cross-validation, and performance on the held-out test set.

- **Model Performance on Training Set**

**Fig. 3:**
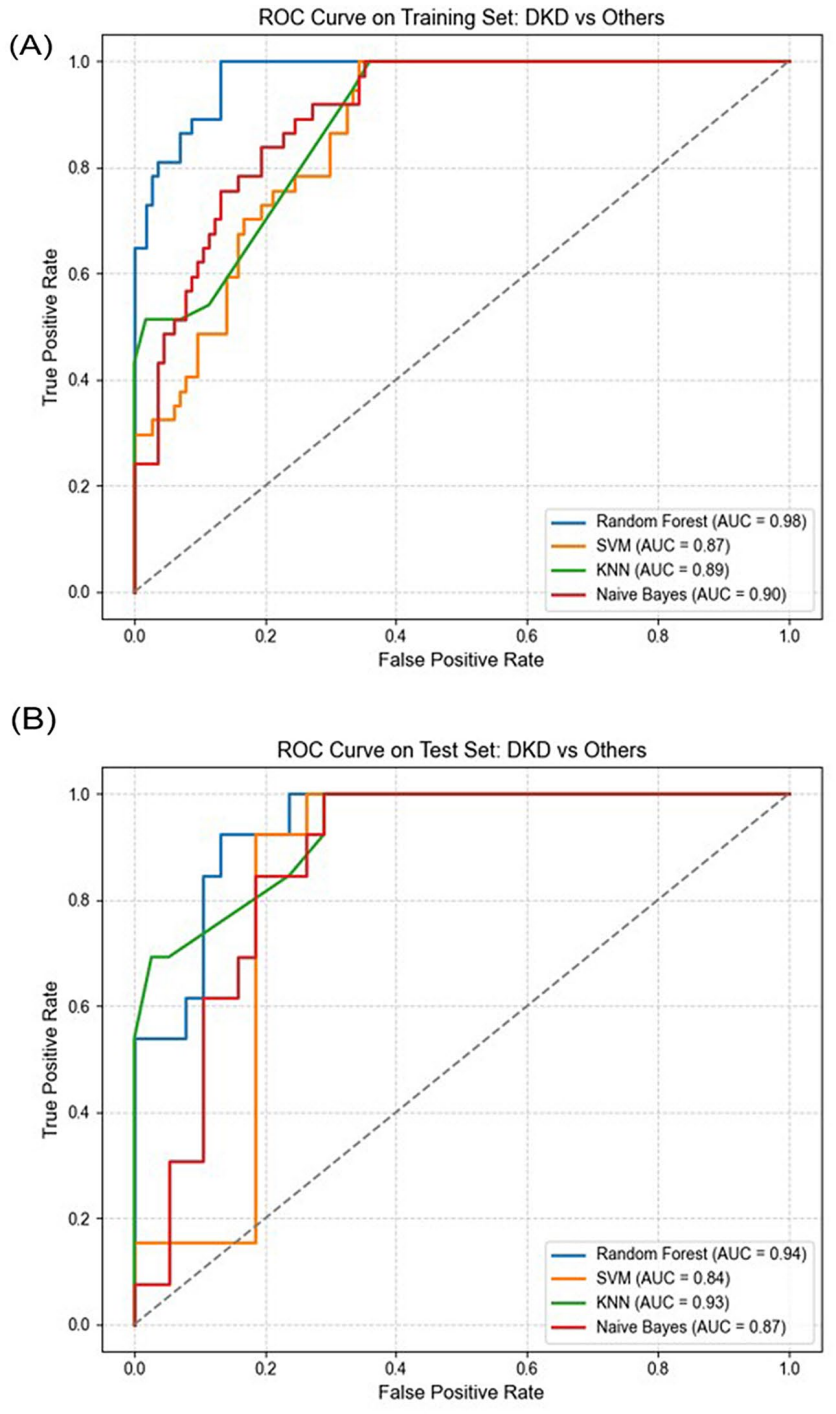
Receiver operating characteristic (ROC) curves of machine learning classifiers (Random Forest, SVM, KNN, and Naïve Bayes) for differentiating DKD from other diagnostic groups. **(A)** ROC curves based on the training set, showing RF with the highest area under the curve (AUC = 0.98), followed by NB (0.90), KNN (0.89), and SVM (0.87). **(B)** ROC curves based on the independent test set, where RF again achieved the best performance (AUC = 0.94), demonstrating strong generalization compared with NB (0.87), KNN (0.93), and SVM (0.84). Abbreviations: RF, Random Forest; KNN, K-Nearest Neighbors; SVM, Support Vector Machine; NB, Naïve Bayes DKD, diabetic kidney disease; AUC, area under the curve; ROC, receiver operating characteristic

All models were initially trained using the training subset, and their performance was evaluated using five key metrics: accuracy, precision, recall, F1-score, and area under the ROC curve (AUC). As presented in Table 3 and Fig. 3, the Random Forest model achieved the highest overall performance, with an accuracy of 0.89, F1-score of 0.88, and the highest AUC of 0.98.

**Table 3:** Comprehensive performance of classification models across training, test, and 5-fold cross-validation datasets

Model	. Performance of Classification Models										Accuracy across 5-fold cross-validation					
	Training Set					Test Set					Fold-1	Fold-2	Fold-3	Fold-4	Fold-5	Mean Accuracy
	Accuracy	Precision	Recall	F1-score	AUC	Accuracy	Precision	Recall	F1-score	AUC						
RF	**0.89**	0.92	0.89	0.88	**0.98**	**0.86**	0.91	0.86	0.86	**0.95**	0.87	0.77	0.83	0.77	0.87	**0.82**
SVM	**0.81**	0.89	0.81	0.77	**0.87**	**0.76**	0.87	0.76	0.71	**0.87**	0.84	0.77	0.77	0.83	0.83	**0.81**
KNN	**0.83**	0.85	0.83	0.83	**0.89**	**0.84**	0.89	0.84	0.84	**0.89**	0.68	0.77	0.67	0.80	0.87	**0.76**
NB	**0.77**	0.79	0.77	0.74	**0.90**	**0.73**	0.85	0.73	0.67	**0.87**	0.68	0.73	0.73	0.80	0.80	**0.75**

The table presents the performance of four classification models (Random Forest, Support Vector Machine, K-Nearest Neighbors, and Naïve Bayes) evaluated on the training and test datasets. Additionally, accuracy from each fold of 5-fold cross-validation is shown, along with the overall mean accuracy, to assess model generalizability and stability

Abbreviations: AUC, area under the ROC curve; RF, Random Forest; SVM, Support Vector Machine, KNN, K-Nearest Neighbors and NB, Naïve Bayes

- **Model Performance on Test Set**

Final model performance was evaluated using the unseen test set. Once again, Random Forest delivered the highest accuracy (0.86) and AUC (0.95), confirming its robustness and strong generalization capabilities (see Table 3 and Fig. 3).

- **Cross-Validation Accuracy**

To evaluate model robustness and reduce overfitting risk, 5-fold cross-validation was conducted on the training set. Random Forest maintained the highest mean accuracy across folds (0.82), reinforcing its consistency and generalizability. Results for all models are summarized in Table 3.

- **Best Performing Model**

**Fig. 4:**
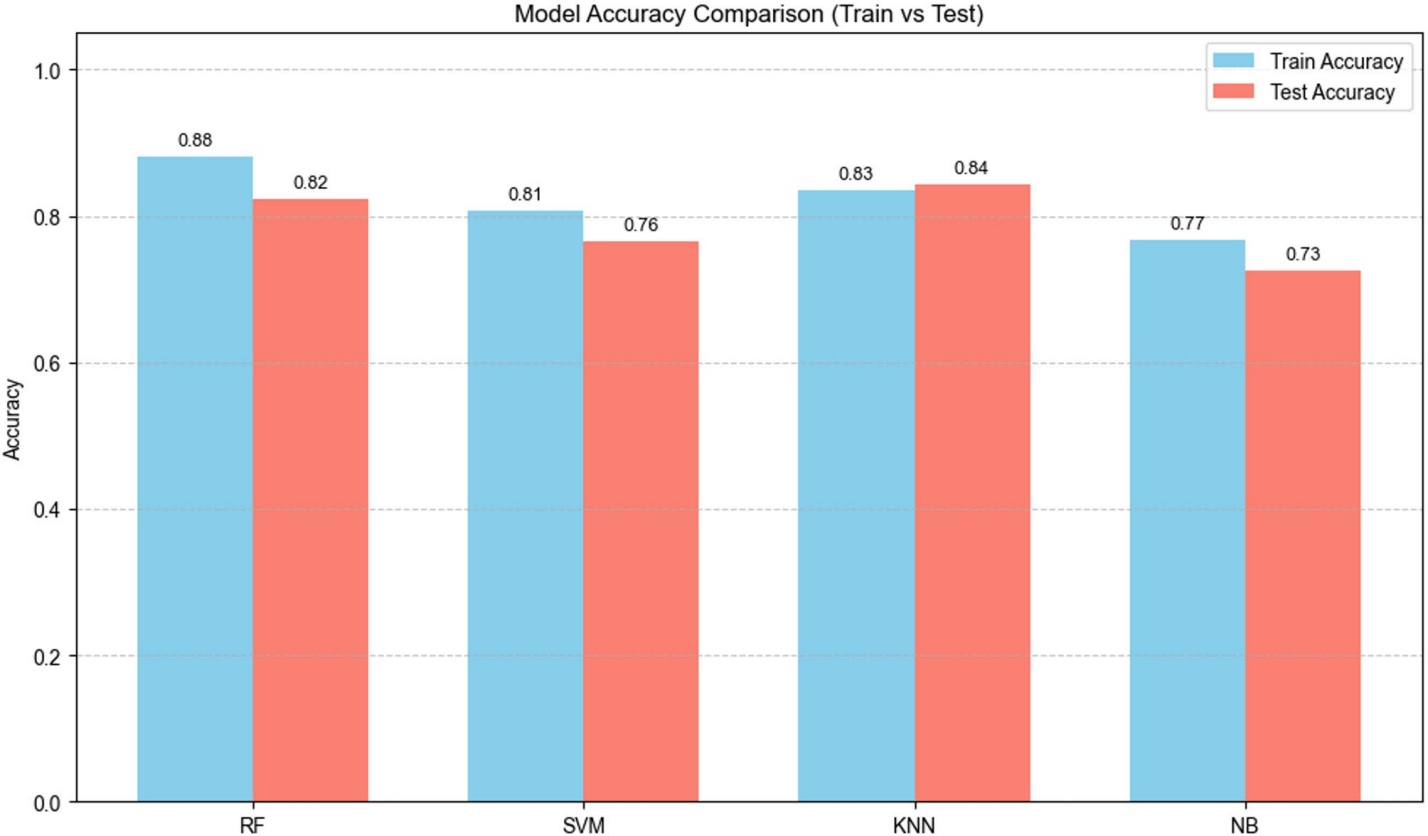
Integrated comparison of classification accuracy across machine learning models after SMOTE balancing. Bar plots compare the performance of four classifiers—RF, SVM, KNN, and NB—on both the training and independent test datasets. The RF model achieved the highest accuracy in both training (0.88) and test (0.82) sets, indicating strong generalization performance and minimal overfitting. These results correspond to the post-SMOTE dataset and align with the final performance metrics presented in Table 3. Abbreviations: RF, Random Forest; KNN, K-Nearest Neighbors; SVM, Support Vector Machine; NB, Naïve Bayes

Considering all evaluation stages—training, cross-validation, and independent testing—the Random Forest classifier demonstrated the most robust and consistent performance. Its high AUC-ROC (0.95) for distinguishing DKD from other classes underscored its clinical utility as a non-invasive triage tool for DKD detection. Figure 4 illustrated the comparative accuracy of all models across both training and test sets, highlighting the superior generalization of RF.

- **Impact of SMOTE on Model Performance**

To evaluate whether the superior performance of Random Forest was attributable to class imbalance, we compared the performance of all four classifiers before and after applying SMOTE (Table 4). Random Forest already achieved reasonable performance in the imbalanced dataset (AUC = 0.76, F1 = 0.53) and further improved after SMOTE (AUC = 0.94, F1 = 0.83). The improvement was mainly in recall and F1-score, indicating better recognition of minority classes, while precision and AUC remained stable. In contrast, SVM, KNN, and Naïve Bayes demonstrated larger fluctuations between before and after SMOTE, suggesting higher sensitivity to class imbalance. Random Forest thus proved robust across both conditions and consistently outperformed the other classifiers. Importantly, Table 4 illustrates the relative effect of SMOTE within training folds, whereas the final independent test set performance is summarized in Table 3, ensuring that the reported superiority of Random Forest is not solely an artifact of oversampling.

**Table 4:** Comparison of model performance before and after SMOTE across four classifiers

Metrics	Before SMOTE	After SMOTE
**Random Forest Model**		
Accuracy	0.62	0.84
AUC	0.76	0.94
Precision	0.58	0.88
Recall	0.49	0.85
F1-score	0.53	0.83
**Support Vector Machine**		
Accuracy	0.62	0.76
AUC	0.57	0.84
Precision	0.73	0.87
Recall	0.70	0.77
F1-score	0.68	0.70
**K-Nearest Neighbors**		
Accuracy	0.59	0.84
AUC	0.74	0.93
Precision	0.71	0.88
Recall	0.71	0.85
F1-score	0.70	0.83
**Naïve Bayse**		
Accuracy	0.62	0.73
AUC	0.67	0.87
Precision	0.59	0.84
Recall	0.46	0.73
F1-score	0.47	0.66

Abbreviations: SMOTE, Synthetic Minority Oversampling Technique, AUC, area under the ROC curve; F1, F1-score (harmonic mean of precision and recall)

- **External Cross-Validation of Bagging Models**

To further strengthen model validation, we employed an external evaluation using a stratified 5-fold cross-validation strategy. In this approach, the dataset was divided into five folds with preserved class distributions. For each iteration, one fold was used exclusively for validation, while the remaining folds were used to train bagging ensemble models based on different classifiers: SVM, KNN, RF, and NB. The procedure was repeated across all folds, and the mean accuracy with standard deviation was computed to provide an unbiased estimate of generalization performance.

To further strengthen model validation, we employed an external evaluation using a stratified 5-fold cross-validation strategy. In this approach, the dataset was divided into five folds with preserved class distributions. For each iteration, one fold was used exclusively for validation, while the remaining folds were used to train bagging ensemble models based on different classifiers: SVM, KNN, RF, and NB. The procedure was repeated across all folds, and the mean accuracy with standard deviation was computed to provide an unbiased estimate of generalization performance.

**Fig. 5:**
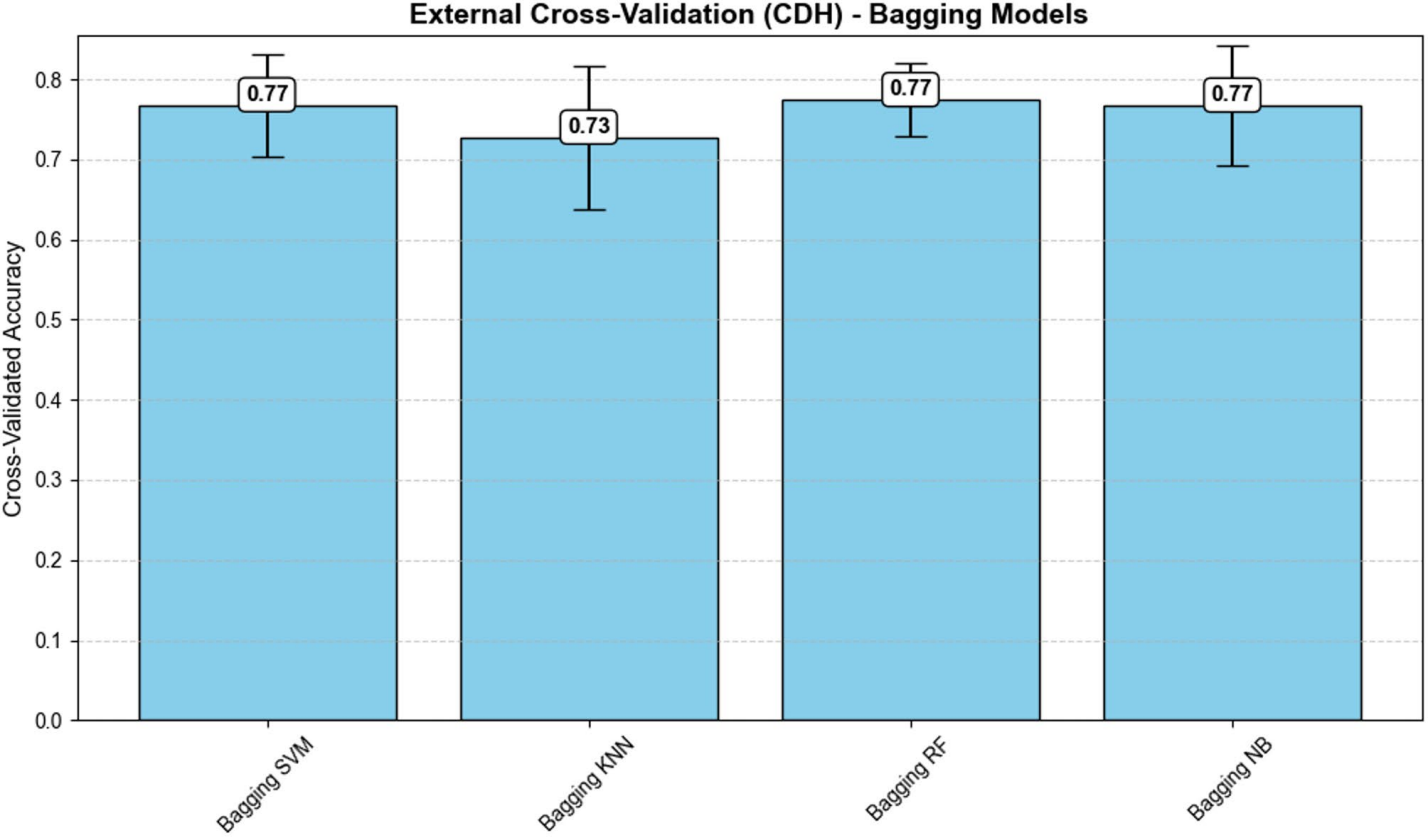
Accuracy of bagging models evaluated via 5-fold stratified cross-validation. Bar plots represent mean cross-validated accuracy, with error bars indicating standard deviations across folds. Bagging Random Forest (RF) achieved the highest mean accuracy (0.77) with the lowest variability, followed by Bagging SVM, NB, and KNN. These results highlight the robustness and stability of the Bagging RF approach in modeling VOC-derived features. Abbreviations: SVM, Support Vector Machine; KNN, K-Nearest Neighbors; RF, Random Forest; NB, Naïve Bayes; VOC, volatile organic compounds

As illustrated in Fig. 5, the Bagging RF model achieved the highest predictive performance (mean accuracy = 0.808, SD = 0.040), combining both accuracy and stability across folds. In contrast, Bagging KNN showed the lowest accuracy (0.728) and highest variability (SD = 0.089), reflecting greater sensitivity to data partitioning. Bagging SVM and Bagging NB yielded comparable mean accuracies (0.768), though SVM demonstrated more consistent results with a smaller variance. Overall, these findings highlight the robustness of the Bagging RF approach, which effectively captured complex interactions in high-dimensional VOC data and maintained stable generalization across repeated evaluations. This external validation provides complementary evidence to the internal cross-validation described earlier, reinforcing the conclusion that Random Forest is the most stable and generalizable classifier in this context.

## Discussion

### Overall model performance

The present study demonstrates that the Random Forest classifier consistently outperformed other models across all evaluation phases, including training, cross-validation, and independent testing. On the training set, RF achieved the highest overall performance, with an accuracy of 0.89, F1-score of 0.88, and an AUC of 0.98 for distinguishing DKD from other diagnostic categories. These metrics underscore the model’s ability to learn complex patterns from VOC signals and translate them into clinically relevant classifications.

Other classifiers showed moderate to acceptable performance. The SVM yielded an AUC of 0.87 with an accuracy of 0.81, indicating good—but less robust—generalization. The KNN provided balanced results (AUC 0.89, accuracy 0.83), while NB, although simple and computationally efficient, performed the lowest (AUC 0.90, accuracy 0.77). Despite its limitations, The NB retained some discriminatory capability, particularly for DKD, suggesting potential utility in resource-limited or rapid-deployment settings.

From a clinical standpoint, accurate detection of DKD remains a high priority, given its prevalence and potential for irreversible renal damage. The binary classification of DKD versus other diagnostic groups revealed that RF achieved the highest AUC across all stages, suggesting that this model is particularly suitable for triage and early identification of DKD in outpatient settings. SVM and KNN may be considered secondary alternatives but were less consistent in terms of cross-validation stability or generalization.

### Why random forest outperformed other models

The superior performance of Random Forest compared with SVM, k-NN, and Naïve Bayes can be attributed to both methodological and dataset-related factors. The VOC-derived features are high-dimensional and non-linear in nature, which favors ensemble-based methods capable of capturing complex interactions. Random Forest aggregates predictions across multiple decision trees, inherently reducing variance and improving stability across cross-validation folds and the independent test set. By contrast, SVM and NB, which assume more linear or parametric relationships, were less effective in modeling this complexity, while k-NN was sensitive to noise and small sample fluctuations. These factors likely explain the consistent advantage of Random Forest in our analysis.

Importantly, validation analyses further reinforced these findings. The before–after SMOTE comparison (Table 4) demonstrated that RF retained superior performance even under imbalanced conditions, with recall and F1-score improving after balancing while AUC and precision remained stable. Furthermore, external cross-validation with bagging ensembles (Fig. 5) confirmed that Bagging RF achieved both the highest mean accuracy and the lowest variability across folds, highlighting its robustness and stability relative to other classifiers. Together, these complementary validation approaches indicate that the superiority of RF is not an artefact of oversampling or a single data split, but rather reflects its methodological suitability for modeling complex, high-dimensional VOC data in DKD detection.

### Why not deep learning in the present study

Although deep learning approaches could, in principle, provide additional modeling capacity for high-dimensional datasets, we deliberately excluded them in this proof-of-concept study. The modest sample size (*n* = 127) posed a high risk of overfitting and limited reproducibility for deep architectures. Moreover, classical ML algorithms offer transparency and interpretability that are essential for early translational research. We therefore prioritized Random Forest and other classical models for this first integration of AI with VOC biosensing, while acknowledging that deep learning should be explored in future studies with larger, externally validated cohorts.

### Added value beyond Single-Signal biomarkers

A critical question arises from our prior research on the same VOC biosensor platform, where chemiresistive signals were measured across four heating cycles using multiple sensors. In that earlier analysis, we identified a single parameter—S2min4, the minimum resistance from sensor 2 during the fourth heating cycle—as a highly discriminative marker. Through traditional statistical comparison of group means, S2min4 alone achieved an AUC approaching 1.0 for distinguishing DKD from other nephropathies [9].

This raises a valid concern: **if a single signal yields near-perfect diagnostic performance**,** what added value does an AI model provide?** While S2min4 demonstrates excellent univariate performance, relying solely on a single parameter may limit diagnostic robustness in real-world applications. Sensor variability, physiologic noise, and overlapping pathologies—such as DKD coexisting with GN—may all degrade its reliability in broader clinical use. Moreover, univariate ROC analysis does not capture interactions or compensatory patterns among other VOC features.

In contrast, machine learning models—particularly ensemble methods like RF—are capable of learning complex, multivariate relationships across high-dimensional feature spaces. These models do not merely select features with the strongest individual associations but instead optimize combinations of features that improve overall model performance. Notably, although S2min4 showed excellent univariate discrimination, it was not consistently ranked among the top features during Random Forest model construction. This highlights a key distinction: **strong individual predictive power does not necessarily translate to additive value in multivariate models.**

Such behavior reflects the theoretical foundation of **Random Forests**, which inherently perform embedded feature selection as part of their ensemble learning process. By evaluating feature contributions across numerous decision trees, the model emphasizes variables that generalize well across subsets of the data, rather than overfitting to highly ranked—but potentially redundant—signals. This principle ensures better robustness and clinical scalability when deployed in real-world settings with complex, overlapping phenotypes [21–24].

### Biological and clinical interpretation

The top 13 features identified through the AI model spanned signals from all five sensors, indicating that discriminative information was not confined to a single source. Each sensor was originally designed to target different classes of VOCs: sensor 1 for methane and iso-butane, sensor 2 for hydrogen and ethanol, sensor 3 for hydrogen sulfide and ammonia, sensor 4 for toluene and propane, and sensor 5 for trimethylamine and methyl-mercaptan. This broad distribution suggests that VOC alterations across diverse metabolic and inflammatory pathways jointly contribute to the biochemical fingerprint of DKD [9].

Among these, sensor 2 (hydrogen, ethanol) appears particularly relevant to DKD, as its signals are likely influenced by oxidative stress and advanced glycation end-products (AGEs)—key processes in DKD pathogenesis—which may explain its greater specificity [9]. In contrast, sensor 3 (hydrogen sulfide, ammonia) contributed multiple discriminatory features across both DKD and non-diabetic nephrotic syndromes, reflecting sensitivity to proteinuria and general glomerular injury rather than DKD-specific mechanisms [9, 15, 16]. Meanwhile, features derived from sensors 1, 4, and 5 (methane, iso-butane, toluene, trimethylamine, methyl-mercaptan) are more consistent with broader CKD progression, fibrosis, and the accumulation of uremic toxins, as supported by prior VOC studies in CKD and interstitial fibrosis [15, 16].

Taken together, this pattern suggests that while sensor 2 may capture DKD-specific metabolic disturbances, the combined contribution of all five sensors reflects both DKD-specific and overlapping pathophysiological processes of proteinuric CKD. Importantly, the AI model does not rely on a single dominant signal but instead extracts diagnostic strength from the distributed pattern of sensor responses.

This biological plausibility reinforces the clinical value of AI-guided VOC analysis—not merely as a statistical tool but as a reflection of the multisystem nature of DKD. From a clinical perspective, the distributed VOC pattern may mirror the multifactorial pathogenesis of DKD, including oxidative stress, altered gut microbiota, uremic toxins, and subclinical inflammation. Accordingly, the AI–VOC biosensor platform may serve as a clinically actionable tool: as a non-invasive screening or triage approach in diabetic clinics, and as a potential strategy to reduce reliance on kidney biopsy by providing greater diagnostic confidence that DKD is distinguished from primary glomerular diseases. Compared with molecular assays such as FRMD3 polymorphism testing [25], or GC–MS–based VOC profiling, the semiconductor biosensor represents a more practical and scalable alternative suitable for routine nephrology practice.

### Limitations

This study has several limitations. First, although the overall cohort was relatively modest, each subject generated a large number of signal-derived features across multiple sensors and heating cycles, providing rich information for training. Nonetheless, the limited sample size remains a constraint. To mitigate imbalance and reduce overfitting, we applied SMOTE within training folds, stratified cross-validation, and bootstrap resampling; however, these techniques cannot fully substitute for larger real-world datasets. Validation was restricted to internal cross-validation and test splitting, and while we additionally performed external stratified cross-validation with bagging ensembles to strengthen robustness, independent external cohorts will ultimately be essential to confirm generalizability.

Second, only classical machine learning algorithms were evaluated. While Random Forest consistently provided the most reliable performance, we deliberately avoided deep learning to reduce the risk of overfitting and to maintain transparency in this proof-of-concept phase. Transfer learning or self-supervised pretraining approaches may also enhance performance once larger VOC datasets become available, and future work with larger multicenter cohorts should explore these directions alongside multimodal integration with clinical biomarkers.

Third, potential confounders such as comorbidities and medication use were not fully stratified due to the secondary nature of the dataset. Finally, although the semiconductor-based VOC biosensor demonstrated promising diagnostic capability, further refinements—including standardized urine sampling, sensor calibration, and long-term reproducibility studies—are required before clinical adoption. Looking ahead, prospective validation in independent and multicenter cohorts will be critical to establish the scalability of this AI–VOC biosensor framework and to define its role as a complementary or triage tool alongside conventional clinical markers. As the present cohort was derived from a single-center Asian population, caution is also warranted in extrapolating these findings globally until confirmed in more diverse settings.

## Conclusion

In conclusion, the Random Forest classifier emerged as the most consistent and accurate model for detecting diabetic kidney disease (DKD) using VOC biosensor signals. Its high AUC and stable performance across evaluation phases provided proof-of-concept evidence that this approach showed *potential as a triage tool for non-invasive DKD* assessment. While it suggested the possibility of reducing reliance on invasive procedures such as kidney biopsy, larger multicenter validation studies were still required before clinical translation.

Although single-signal biomarkers like S2min4 remained informative, the AI-based multivariate approach offered greater robustness and flexibility, particularly in complex or overlapping diagnostic scenarios. Further refinement of the model, including integration with multimodal data and technical standardization, was highlighted as an important next step toward establishing the clinical role of VOC-based AI diagnostics. The robustness of the Random Forest model was further supported by external cross-validation analyses, underscoring its potential for translation to broader clinical practice.

## Supplementary Information

Below is the link to the electronic supplementary material.

Supplementary Material 1

## Data Availability

The datasets generated and/or analyzed during the current study are available from the corresponding author upon reasonable request.

## Abbreviations

DKD: Diabetic Kidney Disease
VOC: Volatile Organic Compounds
UB: Urine Biomarker
ML: Machine Learning
RF: Random Forest
