# Supplementary material for: Artificial intelligence–based diagnosis of diabetic kidney disease using urinary VOC biosensor data
Source: BMC Nephrol. 2025 Nov 26;26:669. doi: 10.1186/s12882-025-04608-z (PMC12659073; doi:10.1186/s12882-025-04608-z)
Supplement: Supplementary file 1 — Supplementary Material 1 [file 12882_2025_4608_MOESM1_ESM.docx]

**Supplementary Data**

**Section I**

**Research Methodology**

This study follows a structured methodology to develop and evaluate a classification model, as illustrated in fig. S1.


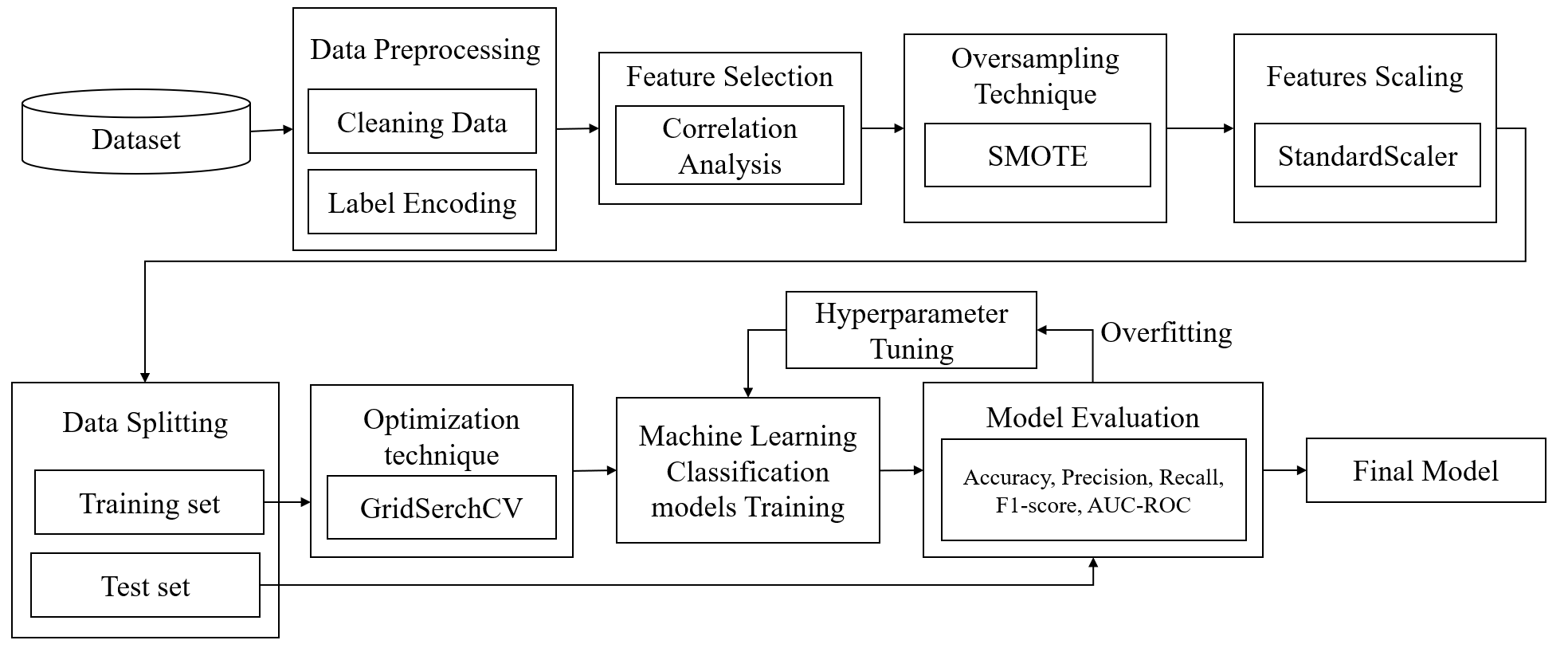


**Fig. S1** Research Methodology workflow

**Figure S1;** **Workflow of the AI-based classification pipeline for DKD**. Data preprocessing included label encoding and quality filtering. Feature selection was conducted using correlation analysis, retaining features with absolute correlation coefficients ≥ 0.80. To address class imbalance, SMOTE oversampling was applied, followed by feature scaling using the StandardScaler method. The dataset was split into training and test sets using stratified sampling. Hyperparameter tuning was performed using GridSearchCV. Multiple machine learning classifiers were trained and evaluated based on accuracy, precision, recall, F1-score, and AUC-ROC to select the final model.

**Abbreviations: AUC-ROC**: Area under the receiver operating characteristic curve**, F1-score**: Harmonic mean of precision and recall**, GridSearchCV**: Grid search with cross-validation**, SMOTE**: Synthetic Minority Over-sampling Technique **StandardScaler**: Standard score normalization method used for feature scaling**.**

**1. Dataset Collection**

The data consisted of 127 samples, each sample containing 90 feature parameters and 1 target class, which is a group of patients, divided into DM 52 samples, DN 50 samples, Nc 8 samples, and No 17 samples.

**2. Data Preprocessing**

**Label Encoding** Categorical variables were transformed into numerical values using label encoding to make the dataset compatible with machine learning algorithms.

**3. Feature Selection**

Feature selection was performed to reduce dimensionality and improve model performance. **Correlation Analysis** Features with high correlation **(above a defined threshold of 0.80)** were selected.

After selecting the parameters with a correlation value greater than 80% both positively and negatively and with a p-value less than 0.01, 127 samples consisting of 13 parameters were obtained, as shown in table S1.

**Table 1. Top 13 VOC-Derived Features with Strongest Correlation to Diagnostic Group** **Classification**

| **Features** | **Correlation** | **p-value** |
| --- | --- | --- |
| Min Sensor 1 Cycle 1 | -0.8039 | 5.4730 x 10^-30^ |
| Min Sensor 2 Cycle 1 | -0.8395 | 6.4774 x 10^-35^ |
| Min Sensor 3 Cycle 2 | -0.8563 | 1.1114 x 10^-37^ |
| Time Sensor 3 Cycle 2 | -0.8331 | 6.1427 x 10^-34^ |
| Min Sensor 3 Cycle 3 | -0.8422 | 2.4738 x 10^-35^ |
| Min Sensor 3 Cycle 4 | -0.8070 | 2.2282 x 10^-30^ |
| Time Sensor 3 Cycle 6 | -0.8079 | 1.7234 x 10^-30^ |
| Min Sensor 4 Cycle 1 | -0.8238 | 1.3246 x 10^-32^ |
| Min Sensor 5 Cycle 1 | -0.8191 | 5.7964 x 10^-32^ |
| Min Sensor 5 Cycle 2 | -0.8231 | 1.6490 x 10^-32^ |
| Gap Sensor 3 Cycle 2 | 0.8530 | 4.1015 x 10^-37^ |
| Gap Sensor 3 Cycle 3 | 0.8437 | 1.4126 x 10^-35^ |
| Gap Sensor 3 Cycle 4 | 0.8203 | 4.0053 x 10^-32^ |

**4. Handling Imbalanced Data**

To address class imbalance in the dataset **SMOTE (Synthetic Minority Over-sampling Technique)** was applied to oversample the minority class, thus improving the model's ability to learn from both classes equally.


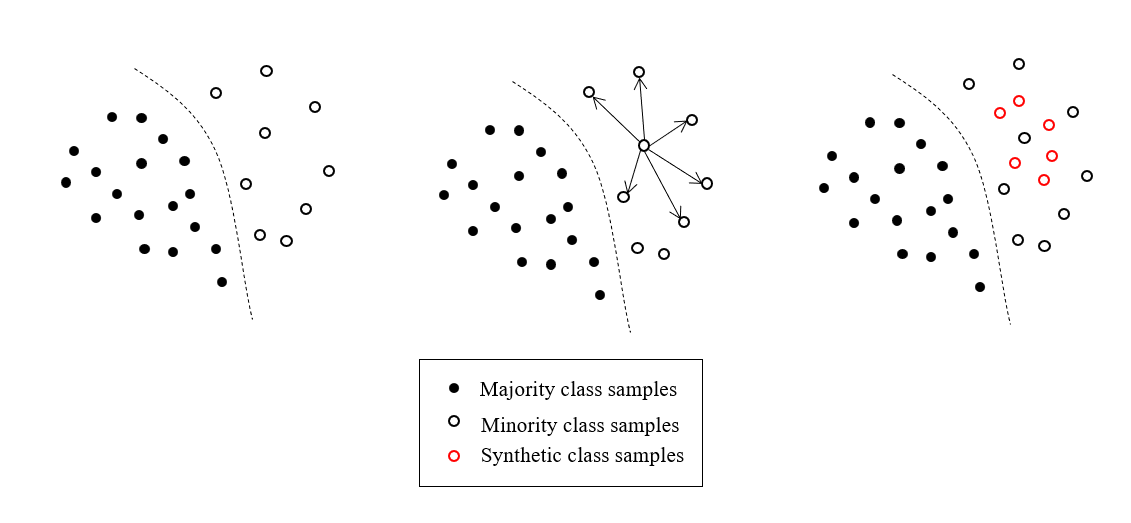


**Fig. S2** Synthetic Minority Over-Sampling Technique

**Figure 2; Conceptual illustration of the SMOTE technique used to address class imbalance**. SMOTE generates synthetic minority samples (red outlines) by interpolating between existing minority class samples (white), helping the model learn more balanced decision boundaries between majority (black) and minority (white) groups.

**Abbreviations: SMOTE,** Synthetic Minority Over-sampling Technique

After performing SMOTE, the data set will consist of 52 DM samples, 50 DN samples, 50 Nc samples, and 50 No samples, totaling 202 samples.

**5. Feature Scaling**

Use **StandardScaler**, a crucial Feature Scaling technique in machine learning, to standardize features by removing the mean and scaling them to unit variance.

$$z= \frac{x-\mu}{\sigma}$$

Where:

$z$ is the standardized value (the new scaled feature value)

$x$ is the original value of the feature

$\mu$ (mu) is the mean of the feature in the training data

$\sigma$ (sigma) is the standard deviation of the feature in the training data

**6. Data Splitting**

The dataset was split into **Training set** and **Test set**, with a split ratio of 75:25. The training set was used for model training and validation, while the test set was reserved for final evaluation.

**7. Optimization Technique**

Hyperparameter tuning was performed using **GridSearchCV**, which systematically explored combinations of hyperparameters to identify the optimal configuration for each classification model.


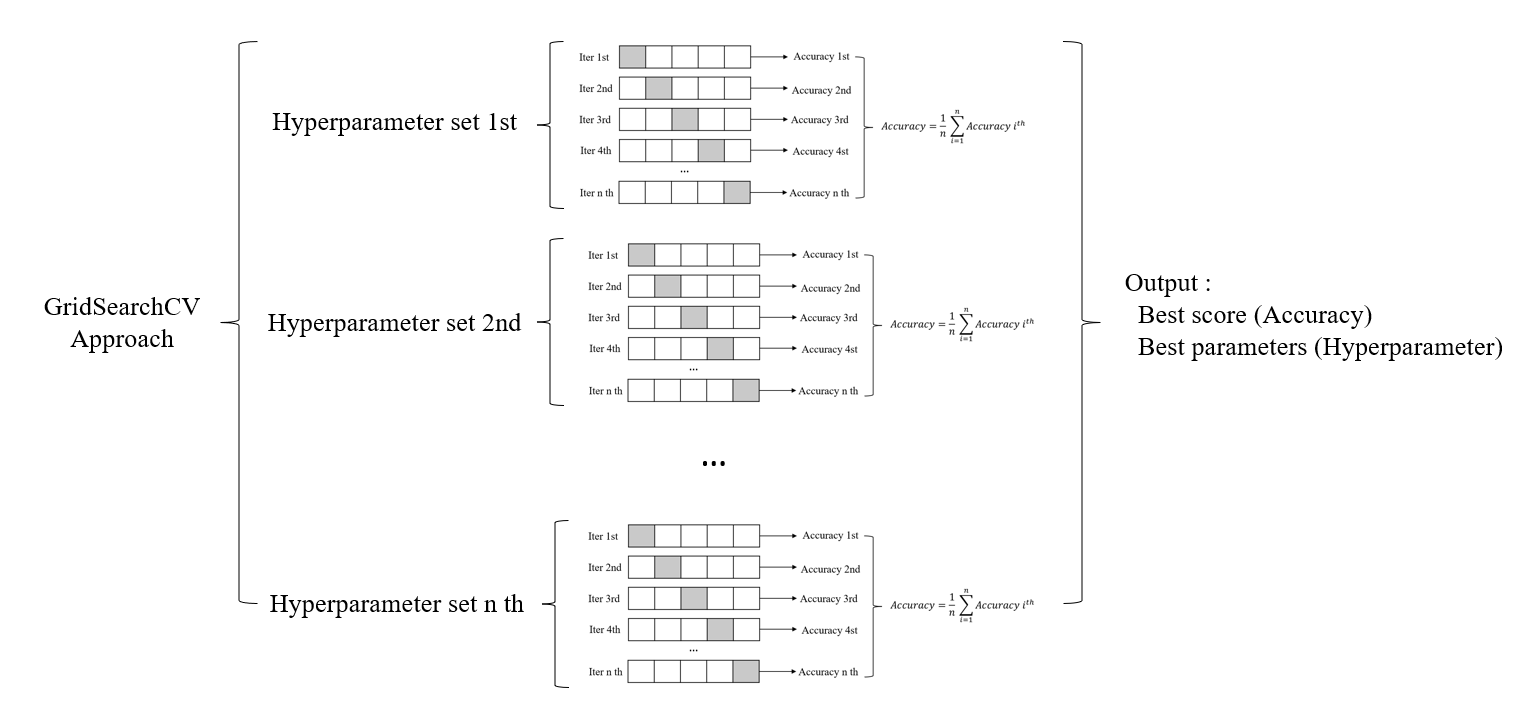


**Fig. S3** GridSearchCV

**Figure 3; Hyperparameter tuning;** GridSearchCV was used to identify optimal hyperparameters by systematically evaluating different parameter combinations using cross-validation within the training set

In conjunction with GridSearchCV, a **5-Fold Cross-Validation** strategy was applied. The training set was split into five subsets; in each iteration, four folds were used for training and one for validation. This process was repeated five times to ensure model robustness and to minimize bias and variance.


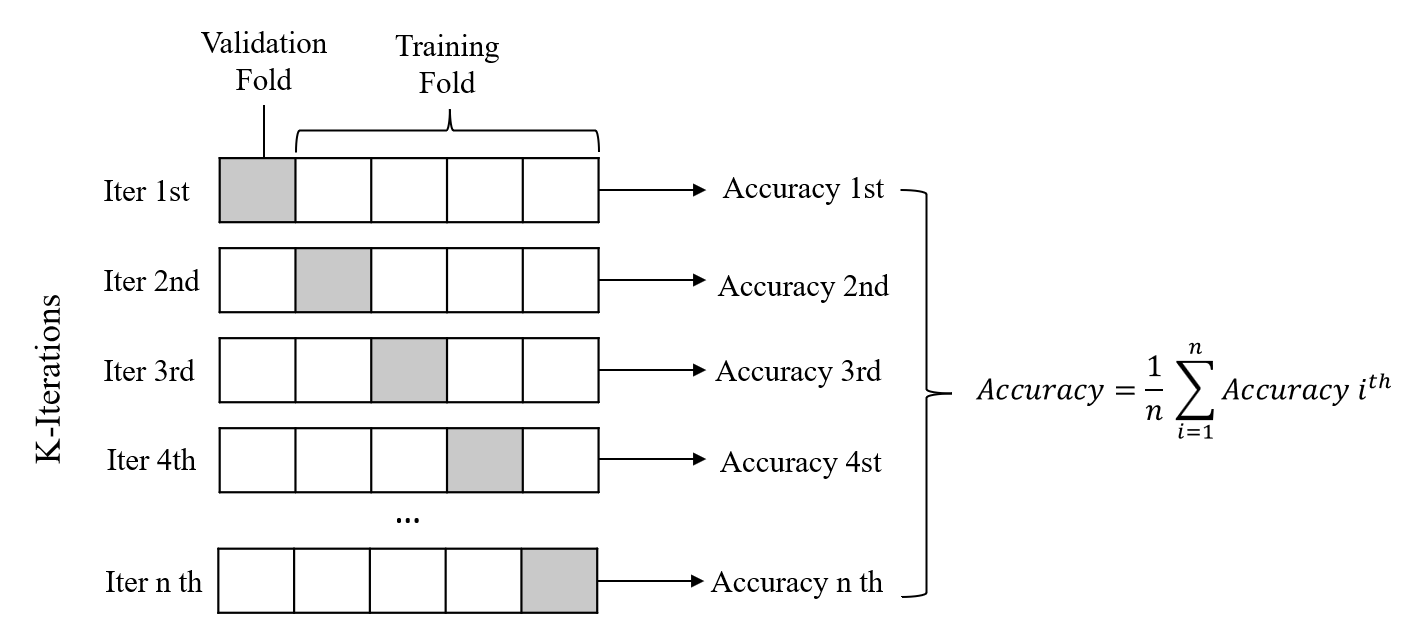


**Fig. S4** K-folds Cross-Validation

**Figure S4; Cross-validation workflow.** A 5-fold cross-validation strategy was applied, in which the training data were split into five equal partitions. Each fold was used once for validation while the remaining four were used for training. The average performance across folds guided hyperparameter selection and minimized the risk of overfitting.

**8. Model Training**

Machine learning classification models, including **Random Forest (RF), Support Vector Machine (SVM), K-Nearest Neighbors (KNN), and , Naïve Bayes** were trained using the training dataset. Each model was trained using the hyperparameters determined during optimization.

**9. Model Evaluation**

The trained models were evaluated using the test dataset. Performance metrics included, Accuracy, Precision, Recall, F1-score, ROC (Receiver Operating Characteristic), and AUC (Area Under the Curve). These performance metrics are able to calculate with equation as follow,

$$Accuracy= \frac{True Negative+True Positive}{Total}$$

$$Precision= \frac{True Positive}{False Positive+True Positive}$$

$$Recall= \frac{True Positive}{False Negative+True Positive}$$

$$F1-score= \frac{2(Precision)(Recall)}{Precision+Recall}$$

When True Positive, True Negative, False Positive and False Negative obtain from Confusion matrix.

|  | Predicted class  (Positive) | Predicted class  (Negative) |
| --- | --- | --- |
| Actual class  (Positive) | True Positive | False Negative |
| Actual class  (Negative) | False Positive | True Negative |

**Fig. S5** Confusion matrix

These metrics provided comprehensive insights into the predictive power and reliability of the models.

Based on evaluation results, the best-performing model with balanced performance across all metrics was selected as the **Final Model** for deployment.

**Section II**

**Results**

The performance of the classification models was evaluated using the test set after completing data preprocessing, feature selection, oversampling with SMOTE, and hyperparameter tuning through 5-fold cross-validation. The models were assessed based on five performance metrics as Accuracy, Precision, Recall, F1-score, and AUC.

**Model Performance Comparison (Training set)**

The following table summarizes the evaluation metrics of the models trained in this study.

**Table S2: Performance of classification models on the training set.**

| **Model** | **Accuracy** | **Precision** | **Recall** | **F1-score** | **AUC** |
| --- | --- | --- | --- | --- | --- |
| Random Forest | **0.8874** | 0.92 | 0.89 | 0.88 | 0.9751 |
| Support Vector Machine | **0.8079** | 0.89 | 0.81 | 0.77 | 0.8672 |
| K-Nearest Neighbors | **0.8344** | 0.85 | 0.83 | 0.83 | 0.8874 |
| Naïve Bayes | **0.7682** | 0.79 | 0.77 | 0.74 | 0.9009 |

To ensure model robustness and reduce the risk of overfitting, a 5-fold cross-validation strategy was applied during the training phase. The results obtained from this process provide valuable insights into the consistency and generalizability of the models across different subsets of the training data.

**Table S3:** **Accuracy across 1-5-fold cross-validation for each model**

| **Model** | **Accuracy** | | | | | **Mean**  **Accuracy** |
| --- | --- | --- | --- | --- | --- | --- |
|  | **Fold-1** | **Fold-2** | **Fold-3** | **Fold-4** | **Fold-5** |  |
| Random Forest | 0.87 | 0.77 | 0.83 | 0.77 | 0.87 | **0.82** |
| Support Vector Machine | 0.84 | 0.77 | 0.77 | 0.83 | 0.83 | **0.81** |
| K-Nearest Neighbors | 0.68 | 0.77 | 0.67 | 0.80 | 0.87 | **0.76** |
| Naïve Bayes | 0.68 | 0.73 | 0.73 | 0.80 | 0.80 | **0.75** |

**Best Performing Model**

Among the evaluated models, **the Random Forest classifier** achieved the best overall performance with an accuracy of 0.89%, F1-score of 0.88 and an AUC of 0.98. This indicates a good balance between sensitivity and specificity, particularly important in handling class imbalance after applying SMOTE.

**Model Performance Comparison (Test set)**

After completing data preprocessing, feature selection, oversampling using SMOTE, and hyperparameter optimization with 5-fold cross-validation, the final models were evaluated using the **unseen test set** to assess their generalization performance.

**Table S4. Accuracy across 5-fold cross-validation and performance of each model of Test set.**

| **Model** | **Accuracy** | **Precision** | **Recall** | **F1-score** | **AUC** |
| --- | --- | --- | --- | --- | --- |
| Random Forest | **0.86** | 0.91 | 0.86 | 0.86 | 0.9494 |
| Support Vector Machine | **0.76** | 0.87 | 0.76 | 0.71 | 0.8672 |
| K-Nearest Neighbors | **0.84** | 0.89 | 0.84 | 0.84 | 0.8874 |
| Naïve Bayes | **0.73** | 0.85 | 0.73 | 0.67 | 0.8725 |

**Generalization Performance**

The test set evaluation reflects the true predictive ability of each model. Among the tested models, the **Random Forest model** achieved the highest performance with an accuracy of 86%, precision of 91%, recall of 86%, and an F1-score of 0.86. This demonstrates the model's ability to maintain robust performance on data it has never seen before, indicating good generalization and low overfitting.

**Section III**

**Result Discussion**

**Overall Model Performance on Training Set**

The training set evaluation showed that the Random Forest model consistently outperformed the other classifiers across all key performance metrics. Accuracy (0.8874), Precision (0.92), Recall (0.89), F1-score (0.88), and AUC of DN compared with Others (0.9751)

This high AUC value indicates that Random Forest is highly effective at distinguishing DN from the other classes. This makes it particularly suitable for clinical decision support where accurate identification of DN is critical.

The other models performed as follows SVM is Moderate performance (AUC: 0.8672, Accuracy: 0.8079). KNN is Balanced performance (AUC: 0.8874, Accuracy: 0.8344). Naïve Bayes is Lowest among the group (AUC: 0.9009, Accuracy: 0.7682), though it still showed decent AUC, suggesting it maintains some discriminative power specifically for DN.

**Cross-Validation for Robustness**

A 5-fold cross-validation strategy was employed to evaluate model stability and generalizability. The average cross-validated accuracy showed consistent trends with the training performance.

**Table S5: Accuracy across 5-fold cross-validation of each model.**

| **Model** | **Mean CV Accuracy** |
| --- | --- |
| **Random Forest** | **0.82** |
| Support Vector Machine | **0.81** |
| K-Nearest Neighbors | **0.76** |
| Naïve Bayes | **0.75** |

Despite slight drops from training accuracy (expected due to overfitting control), **Random Forest retained the highest average accuracy**, demonstrating robustness across data partitions.

**Test Set Performance**

The model performance on the unseen test data confirmed the training and cross-validation trends.

**Table S6: Accuracy across 5-fold cross-validation and performance of each model.**

| **Model** | **Accuracy** | **AUC (DN vs. Others)** |
| --- | --- | --- |
| **Random Forest** | **0.86** | **0.9751** |
| K-Nearest Neighbors | 0.84 | 0.8874 |
| Support Vector Machine | 0.76 | 0.8672 |
| Naïve Bayes | 0.73 | 0.8725 |

Random Forest again achieved the **highest AUC (0.9751)**, solidifying its capability in **discriminating DN from other conditions**. Interestingly, despite lower overall accuracy, Naïve Bayes retained a competitive AUC, suggesting that it may still be useful in scenarios where computational simplicity is prioritized.

**Clinical Relevance (Focusing on DN Classification)**

Since Diabetic Nephropathy (DN) is a critical complication of diabetes with severe implications, the ability to accurately detect it is paramount. The AUC metric, used specifically here to assess binary classification of DN vs. Others, provides a focused view on the model’s sensitivity and specificity for this condition.

Random Forest, with its consistently high AUC across training (0.9751), validation, and test phases, emerges as the most clinically reliable model for identifying DN.

SVM and KNN follow as secondary options but fall short in either generalization (SVM) or stability (KNN).

Naïve Bayes, although simple and fast, may be more prone to variability, especially in multiclass contexts.


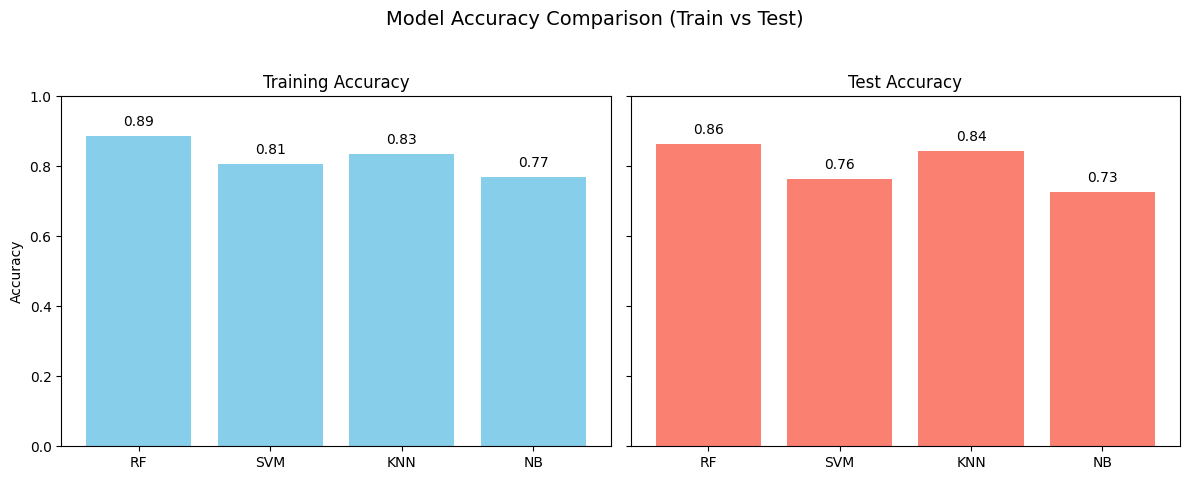


**Fig. S3** Model accuracy comparison on Training set and Test set

**Figure. S3 Comparison of classification accuracy across models on training and test sets**. Bar plots compare the accuracy of four machine learning models—RF, SVM, KNN, and NB—on both the training and independent test datasets. The RF model achieved the highest accuracy in both settings, indicating strong generalization performance and minimal overfitting.

**Abbreviations:** RF, Random Forest; KNN, K-Nearest Neighbors; SVM, Support Vector Machine; NB, Naïve Bayes

**
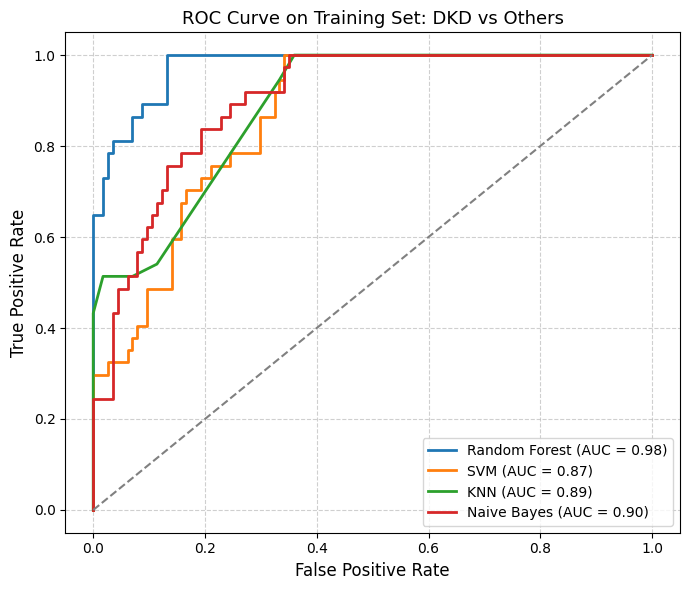
**

**Fig. S4** ROC curve on Training set

**Fig. S4 ROC curve on Training set:** ROC curves based on the training set. RF demonstrated the highest area under the curve (AUC = 0.98), followed by NB, KNN, and SVM.

**Abbreviations:** RF, Random Forest; KNN, K-Nearest Neighbors; SVM, Support Vector Machine; NB, Naïve Bayes DKD, diabetic kidney disease; AUC, area under the curve; ROC, receiver operating characteristic.

**
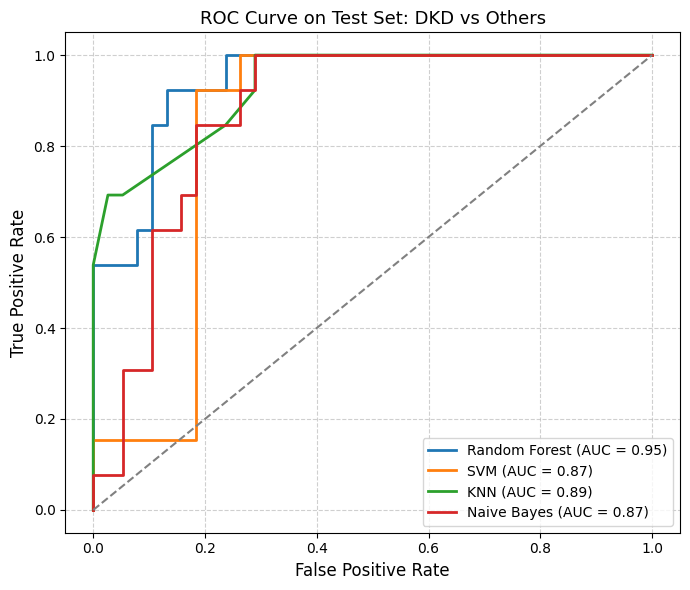
**

**Fig. S5** ROC curve on Test set

**Fig. S5 ROC curve on Test set:** RF maintained the best discriminative performance with an AUC of 0.95, indicating strong generalization ability in distinguishing DKD from other diagnostic groups.

**Abbreviations:** RF, Random Forest; KNN, K-Nearest Neighbors; SVM, Support Vector Machine; NB, Naïve Bayes DKD, diabetic kidney disease; AUC, area under the curve; ROC, receiver operating characteristic.

**Conclusion**

The findings underscore the strength of the **Random Forest classifier** in multi-class settings, particularly when a **highly sensitive and specific model for detecting DN** is required. Its superior AUC and robustness across training, validation, and testing phases affirm its suitability for medical diagnostic applications.

Further refinement using feature selection or ensemble learning could enhance even these results, but based on current metrics, **Random Forest offers the best balance of accuracy, precision, recall, and DN-specific discrimination**.

**Section IV**

**Appendix**

**1. Model Evaluation**

**1.1 Random Forest**

**
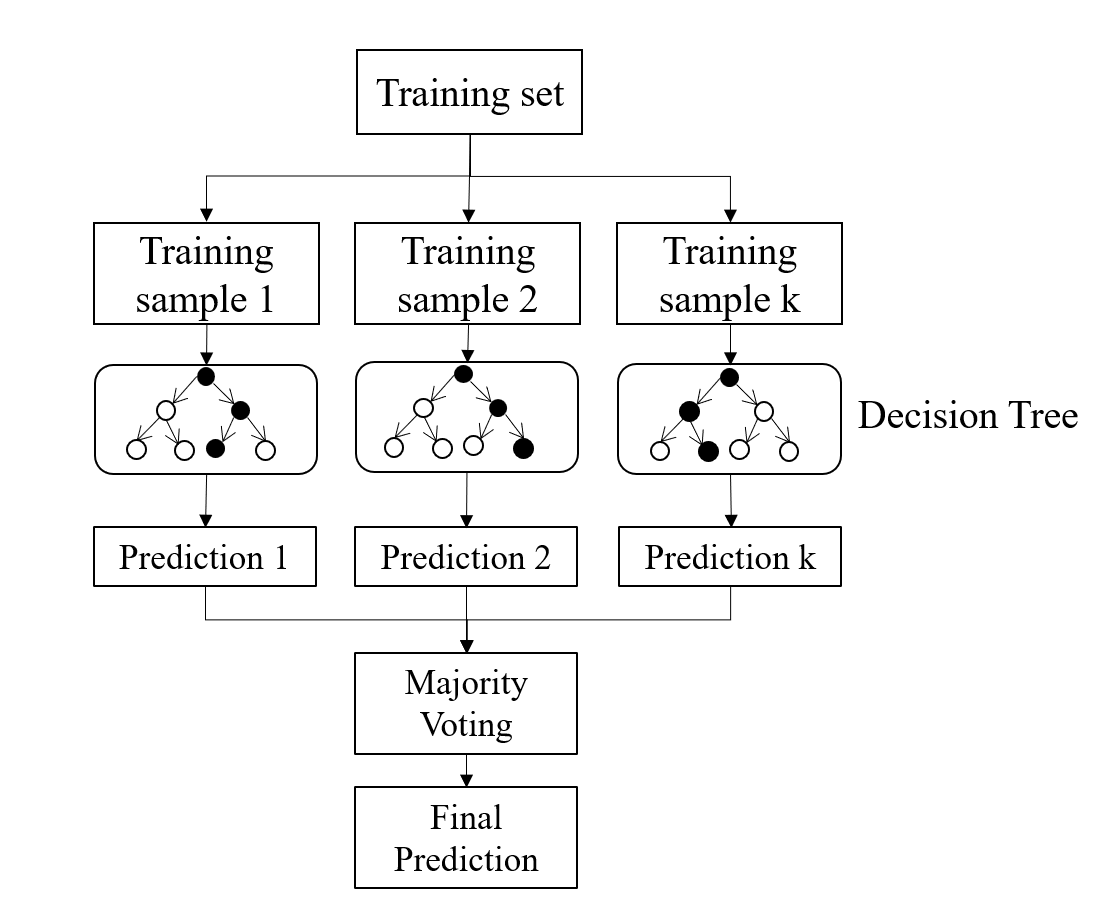
**

In the **Random Forest (RF)** model, three key parameters—**n_estimators, criterion, and max_depth**—play a crucial role in shaping model performance and generalization. The **n_estimators** parameter defines the number of decision trees in the ensemble; increasing this typically enhances model stability and accuracy, though with diminishing returns and increased computation. The **criterion** determines how splits in each tree are evaluated, commonly using either the Gini impurity ('gini') or information gain based on entropy ('entropy'), where the latter may offer better performance on imbalanced data. The **max_depth** parameter sets the maximum number of levels for each tree, acting as a control on model complexity: shallower trees (lower depth) reduce overfitting, while deeper trees capture more detail but may lead to overfitting if not properly tuned. Together, these parameters must be carefully optimized to balance model accuracy, interpretability, and computational efficiency.

- - 1. **GridSearchCV Parameters**

| **Hyperparameters** | **Values** |
| --- | --- |
| n_estimators | 200, 210, 220, 230, 240, 250 |
| criterion | gini, entropy, log_loss |
| max_depth | 3, 5, 7, 9 |

From total 72 model’s hyperparameter sets

Best Parameters: {'criterion': 'entropy', 'n_estimators': 240, 'max_depth': 5}

**Best Accuracy: 0.7880**

- - 1. **Model Performance on Training set**

| Class | Precision | Recall | F1-score | Support Instance |
| --- | --- | --- | --- | --- |
| DM | 0.72 | 1.00 | 0.84 | 41 |
| DN | 1.00 | 0.57 | 0.72 | 37 |
| NC | 0.97 | 1.00 | 0.99 | 37 |
| No | 1.00 | 0.97 | 0.99 | 36 |
| All  (weighted avg) | 0.92 | 0.89 | 0.88 | 151 |

**Training Accuracy: 0.8874**

model Cross-validation scores (5 folds): 0.87096774, 0.76666667, 0.83333333, 0.76666667, 0.86666667

model Mean CV Accuracy: 0.8209

model Std of CV Accuracy: 0.0461


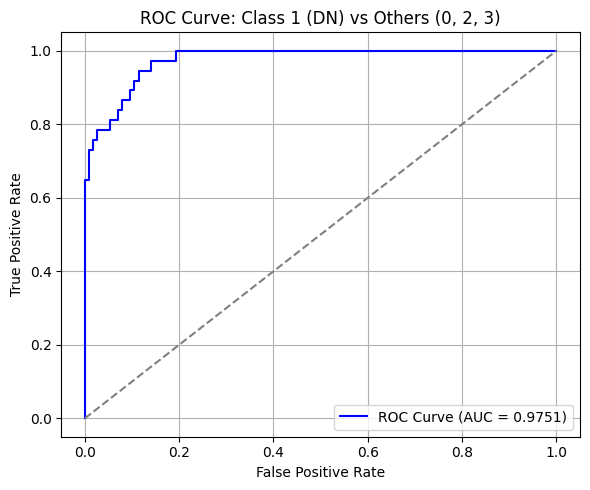


- - 1. **Model Performance on Test set**

**Table S9 Model Performance on Test set**

| Class | Precision | Recall | F1-score | Support Instance |
| --- | --- | --- | --- | --- |
| DM | 0.65 | 1.00 | 0.79 | 11 |
| DN | 1.00 | 0.54 | 0.70 | 13 |
| NC | 0.93 | 1.00 | 0.96 | 13 |
| No | 1.00 | 0.93 | 0.96 | 14 |
| All  (weighted avg) | 0.91 | 0.86 | 0.86 | 51 |

**Test Accuracy: 0.86**


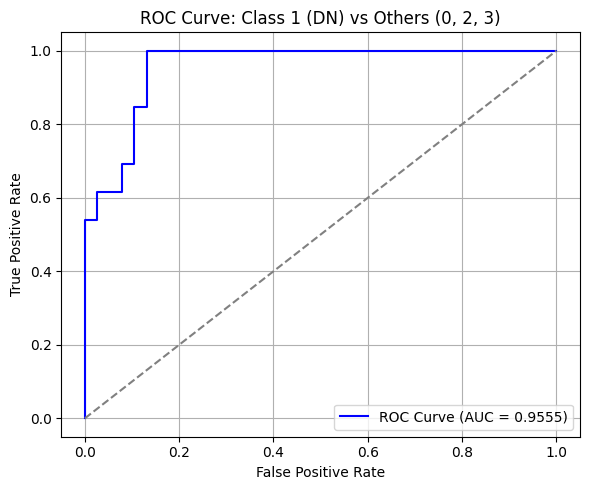


**1.1.4 Confusion Matrix of Random Forest (Test set)**


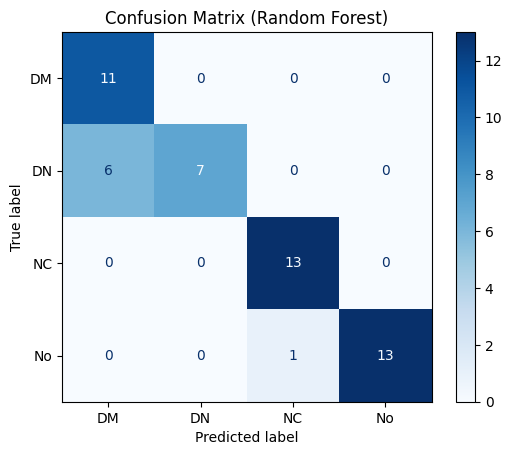


**1.1.5 Learning Curve**


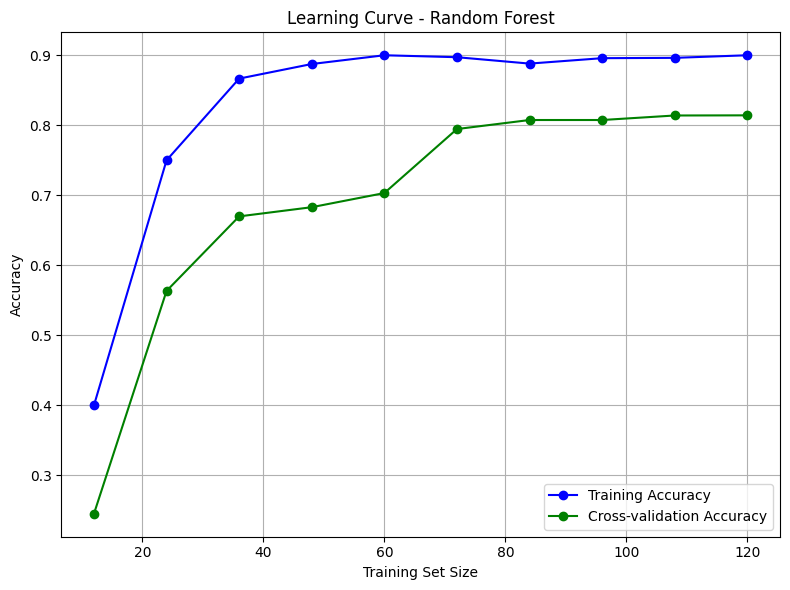


**Learning curve RF**

**1.1.6 Loss Curve**


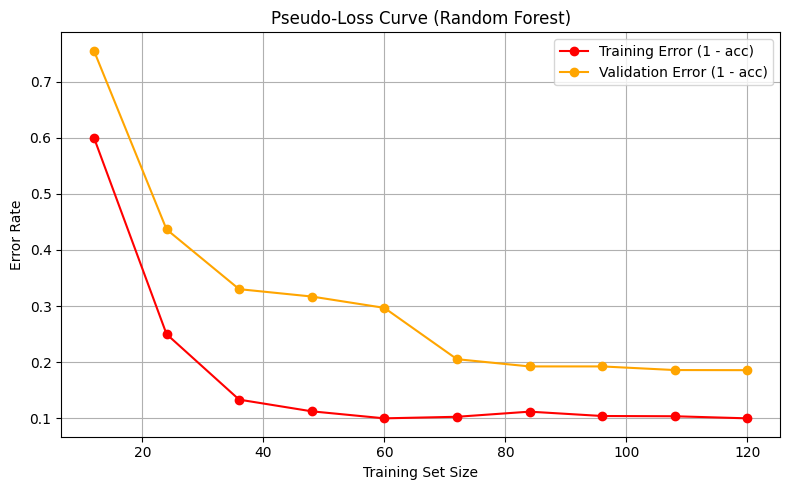


**Pseudo-loss curve RF**

**1.2 Support Vector Machine**

**
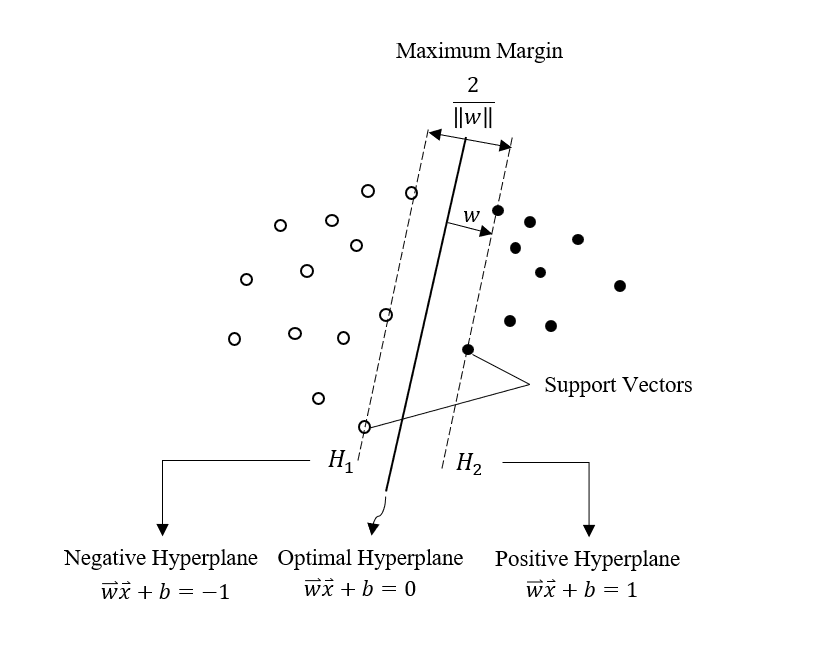
**

**SVM**

In a **Support Vector Machine (SVM)**, the parameter **C** controls the trade-off between achieving a low trai

ning error and a large margin. A small C value allows the model to choose a larger-margin hyperplane, even if it misclassifies more points (soft margin), thus promoting generalization. A large C value aims to classify all training examples correctly, which can lead to overfitting. The **kernel** **function** determines how the input data is transformed into a higher-dimensional space to handle non-linear relationships; common kernels include linear, polynomial, radial basis function (RBF), and sigmoid. The **gamma** parameter, relevant for RBF, polynomial, and sigmoid kernels, defines how far the influence of a single training example reaches: a low gamma means ‘far’ and leads to smoother decision boundaries, while a high gamma means ‘close’ and can cause overfitting by making the boundary fit too tightly to the data.

**1.2.1 GridSearchCV Parameters**

| **Hyperparameters** | **Values** |
| --- | --- |
| C | 0.01, 0.1, 1, 5, 10, 15, 20 |
| Kernel | linear, rbf, poly |
| gamma | scale, auto |

From total 42 model’s hyperparameter sets

Best Parameters: {'C': 10, 'gamma': 'scale', 'kernel': 'linear'}

**Best Accuracy: 0.8211**

**1.2.2 Model Performance on Training set**

| Class | Precision | Recall | F1-score | Support Instance |
| --- | --- | --- | --- | --- |
| DM | 0.59 | 1.00 | 0.74 | 41 |
| DN | 1.00 | 0.22 | 0.36 | 37 |
| NC | 1.00 | 1.00 | 1.00 | 34 |
| No | 1.00 | 1.00 | 1.00 | 39 |
| All  (weighted avg) | 0.89 | 0.81 | 0.77 | 151 |

**Training Accuracy: 0.8079**

model Cross-validation scores (5 folds): 0.83870968, 0.76666667, 0.76666667, 0.83333333, 0.83333333

model Mean CV Accuracy: 0.8077

model Std of CV Accuracy: 0.0336


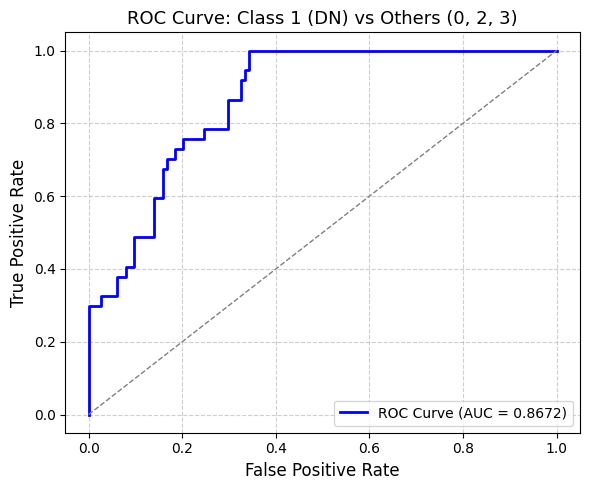


**ROC-AUC of Training set**

**1.2.3 Model Performance on Test set**

| Class | Precision | Recall | F1-score | Support Instance |
| --- | --- | --- | --- | --- |
| DM | 0.48 | 1.00 | 0.65 | 11 |
| DN | 1.00 | 0.08 | 0.14 | 13 |
| NC | 1.00 | 1.00 | 1.00 | 16 |
| No | 1.00 | 1.00 | 1.00 | 11 |
| All  (weighted avg) | 0.89 | 0.76 | 0.71 | 51 |

**Test Accuracy: 0.78**


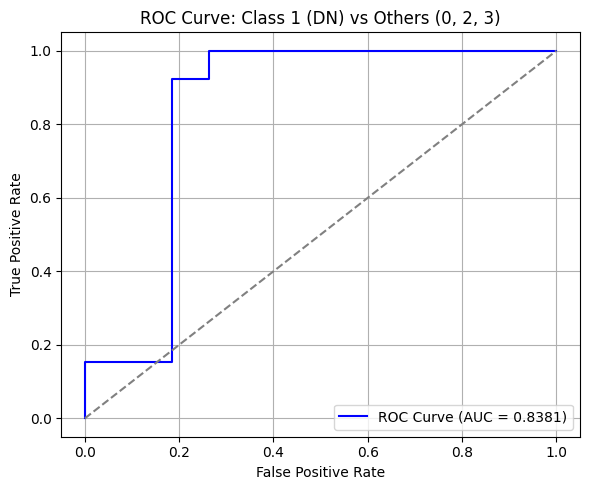


**ROC-AUC of Test set**

**1.2.4 Confusion Matrix of Support Vector Machine (Test set)**


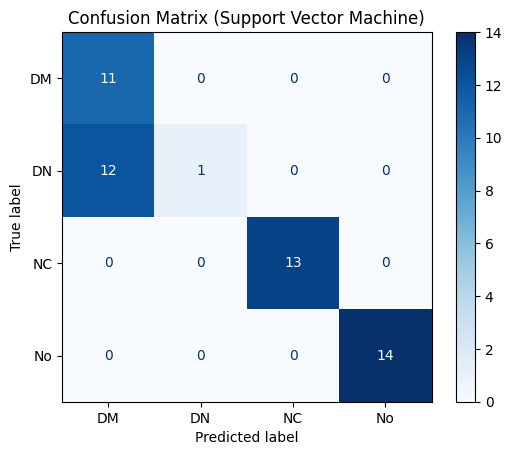


**1.2.5 Learning Curve**


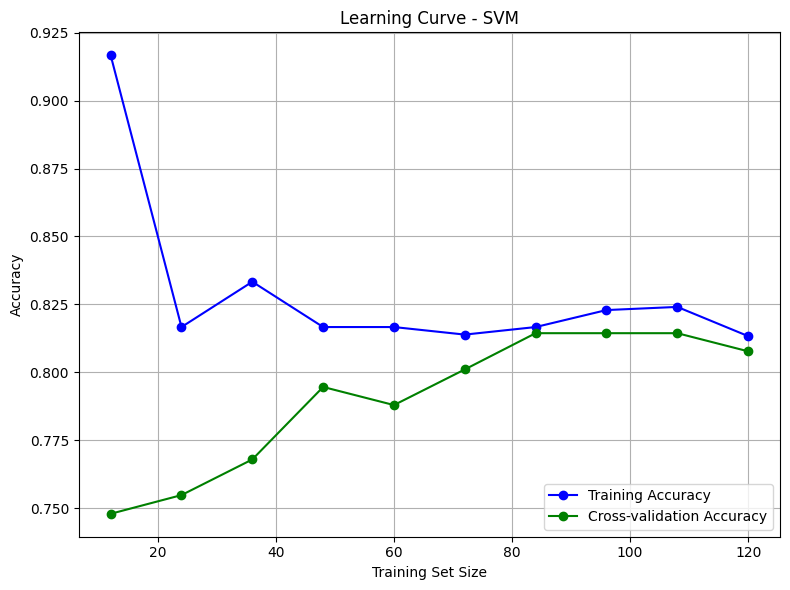


**1.2.6 Loss Curve**


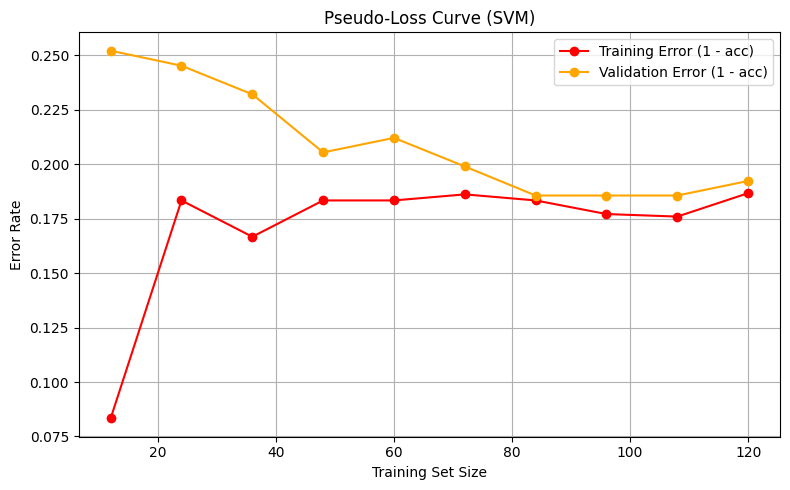


**1.3 K-Nearest Neighbors (KNN)**

**
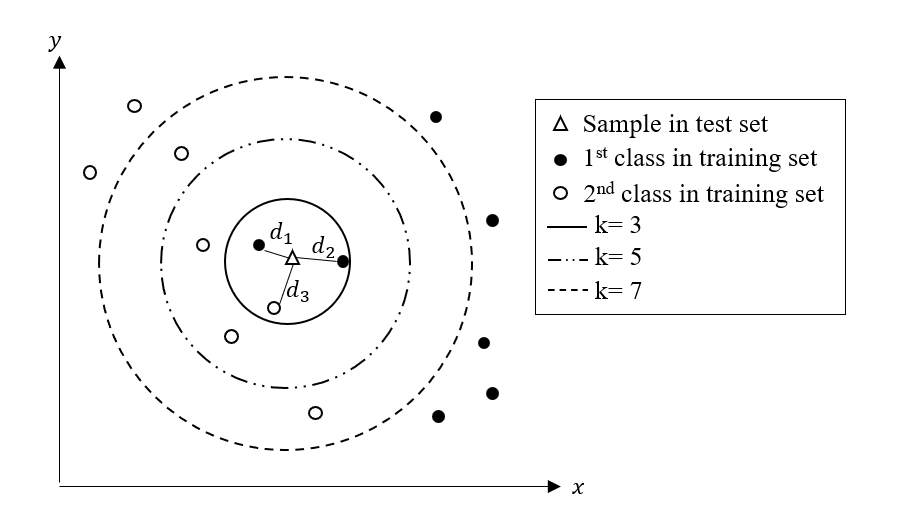
**

In the **k-Nearest Neighbors (KNN**) model, the **n_neighbors** parameter defines the number of nearest training samples considered when classifying a test point; a small value like k=3 leads to a more flexible model that can capture local structure but may be sensitive to noise, while a larger k smooths the decision boundary and reduces variance. The **weights** parameter determines how each of the k neighbors contributes to the classification: 'uniform' treats all neighbors equally, while 'distance' gives more weight to closer neighbors, making predictions more sensitive to nearby points. The **p** parameter sets the power for the Minkowski distance used to compute closeness: p=2 corresponds to the standard Euclidean distance, while p=1 uses Manhattan distance, allowing the model to adapt to different geometries of the feature space.

**1.3.1 GridSearchCV Parameters**

| **Hyperparameters** | **Values** |
| --- | --- |
| n_neighbors | 3, 5, 7, 9, 11, 13 |
| weights | uniform, distance |
| p | Manhattan distance, Euclidean distance |

From total 24 model’s hyperparameter sets

Best Parameters: {'n_neighbors': 7, 'p': ‘Euclidean distance’, 'weights': 'distance'}

**Best Accuracy: 0.7553**

**1.3.2 Model Performance on Training set**

| Class | Precision | Recall | F1-score | Support Instance |
| --- | --- | --- | --- | --- |
| DM | 0.67 | 0.88 | 0.76 | 41 |
| DN | 0.79 | 0.51 | 0.62 | 37 |
| NC | 0.95 | 1.00 | 0.97 | 34 |
| No | 1.00 | 0.94 | 0.97 | 39 |
| All  (weighted avg) | 0.85 | 0.83 | 0.83 | 151 |

**Training Accuracy: 0.8344**

model Cross-validation scores (5 folds): 0.67741935, 0.76666667, 0.66666667,0.8, 0.86666667

model Mean CV Accuracy: 0.7555

model Std of CV Accuracy: 0.0754


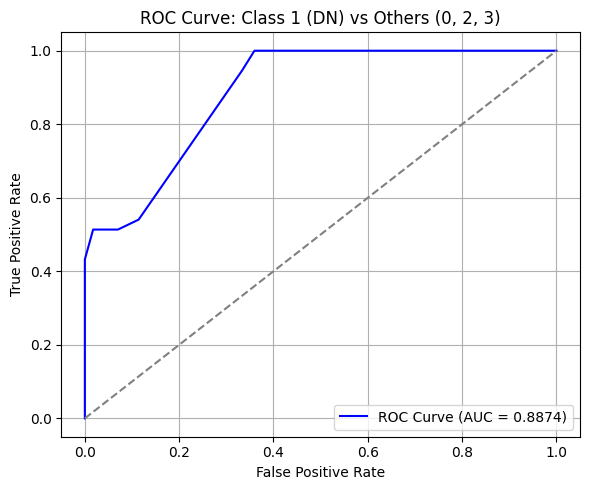


**ROC-AUC of Training set**

**1.3.3 Model Performance on Test set**

| Class | Precision | Recall | F1-score | Support Instance |
| --- | --- | --- | --- | --- |
| DM | 0.65 | 1.00 | 0.79 | 11 |
| DN | 1.00 | 0.54 | 0.70 | 13 |
| NC | 0.87 | 1.00 | 0.93 | 16 |
| No | 1.00 | 0.86 | 0.92 | 11 |
| All  (weighted avg) | 0.89 | 0.84 | 0.84 | 51 |

**Test Accuracy: 0.84**


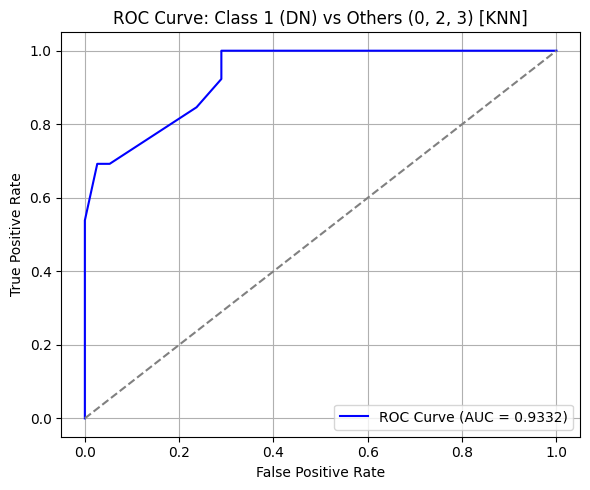


**ROC-AUC of Test set**

**1.3.4 Confusion Matrix of K-Nearest Neighbors (Test set)**


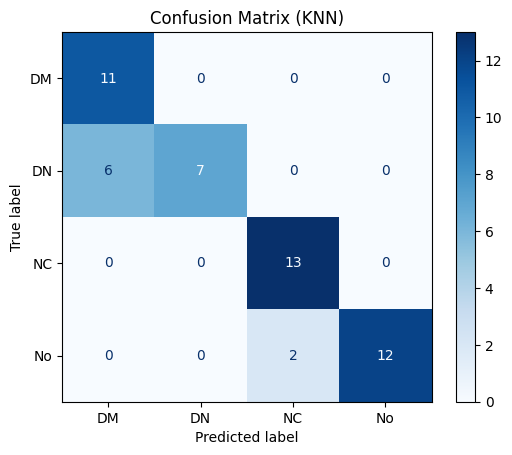


**1.3.5 Learning Curve**


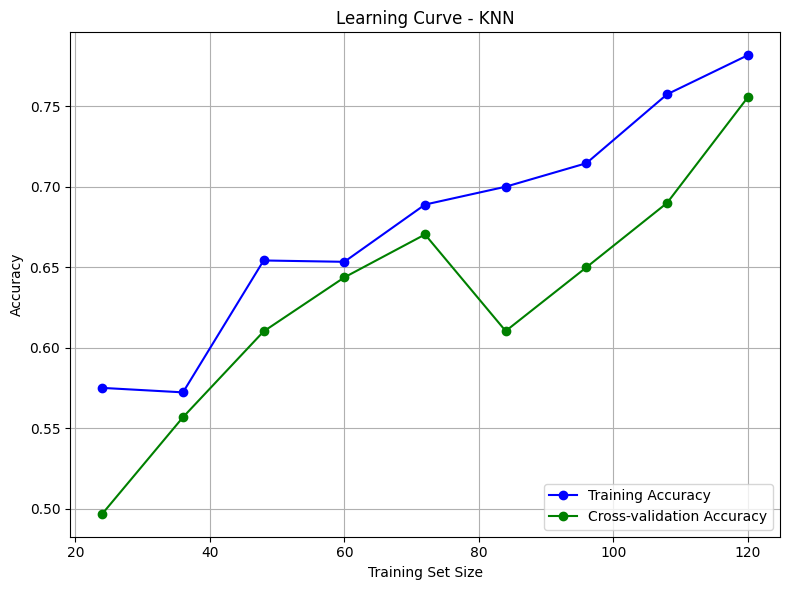


**1.3.6 Loss Curve**


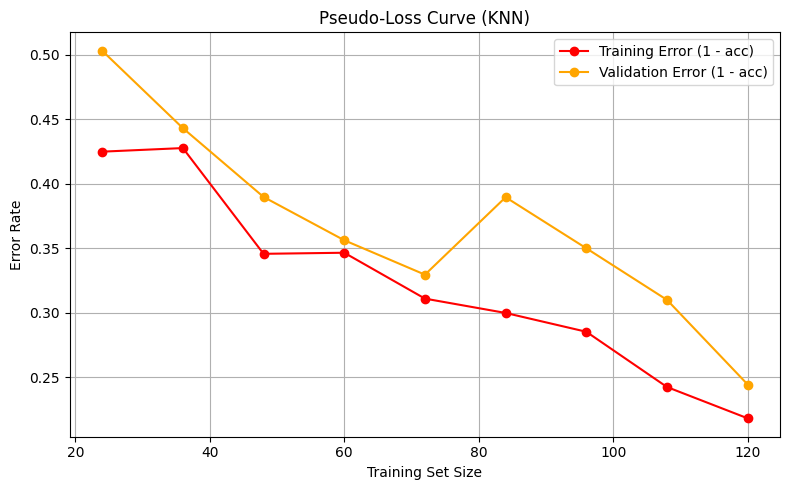


**1.4 Naive Bayse**

$$P\left( A | B \right)=\frac{P\left( B | A \right)P(A)}{P(B)}$$

In **the Naive Bayes classifier**, particularly the Gaussian Naive Bayes variant, the **var_smoothing** parameter is a small positive number added to the variance of each feature to prevent division by zero or extremely small values during likelihood computation. This smoothing is crucial when dealing with features that have very low variance, which could otherwise make the model numerically unstable. By default, var_smoothing is typically set to a small value like 1e-9, and increasing it can make the model more robust to noisy or sparse data, while decreasing it can increase sensitivity but may lead to overfitting or numerical errors.

**1.4.1 GridSearchCV Parameters**

| **Hyperparameters** | **Values** |
| --- | --- |
| var_smoothing | 1e-9, 1e-8, 1e-7, 1e-6 |

From total 4 model’s hyperparameter sets

Best Parameters: {'var_smoothing': 1e-09}

**Best Accuracy: 0.7877**

**1.4.2 Model Performance on Training set**

| Class | Precision | Recall | F1-score | Support Instance |
| --- | --- | --- | --- | --- |
| DM | 0.57 | 0.90 | 0.70 | 41 |
| DN | 0.69 | 0.24 | 0.36 | 37 |
| NC | 0.93 | 1.00 | 0.96 | 34 |
| No | 1.00 | 0.92 | 0.96 | 39 |
| All  (weighted avg) | 0.79 | 0.77 | 0.74 | 151 |

**Training Accuracy: 0.7682**

model Cross-validation scores (5 folds): 0.67741935, 0.73333333, 0.73333333, 0.8, 0.8

model Mean CV Accuracy: 0.7488

model Std of CV Accuracy: 0.0465


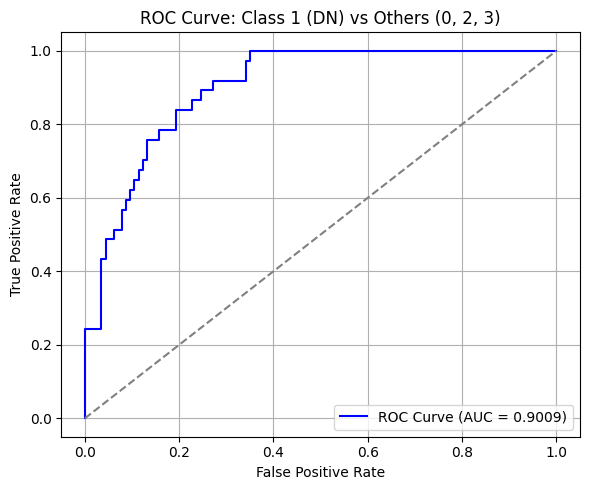


**ROC-AUC of Training set**

**1.4.3 Model Performance on Test set**

| Class | Precision | Recall | F1-score | Support Instance |
| --- | --- | --- | --- | --- |
| DM | 0.48 | 1.00 | 0.65 | 11 |
| DN | 1.00 | 0.08 | 0.14 | 13 |
| NC | 0.87 | 1.00 | 0.93 | 16 |
| No | 1.00 | 0.86 | 0.92 | 11 |
| All  (weighted avg) | 0.85 | 0.73 | 0.67 | 51 |

**Test Accuracy: 0.73**


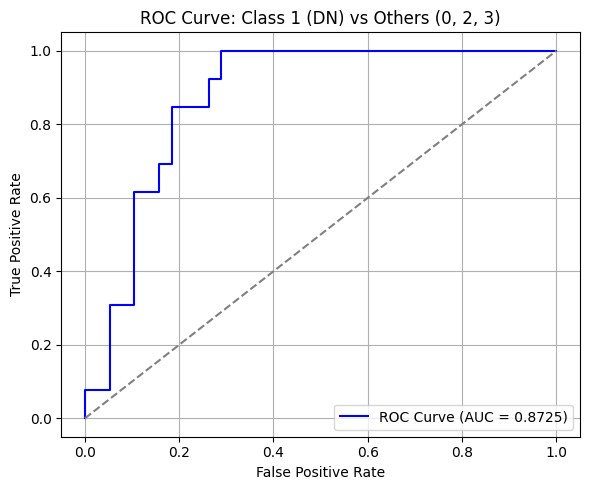


**ROC-AUC of Test set**

**1.4.4 Confusion Matrix of Naïve Bayse (Test set)**


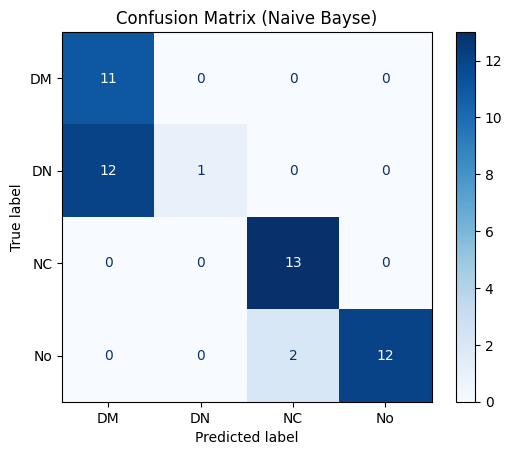


**1.4.5 Learning Curve**


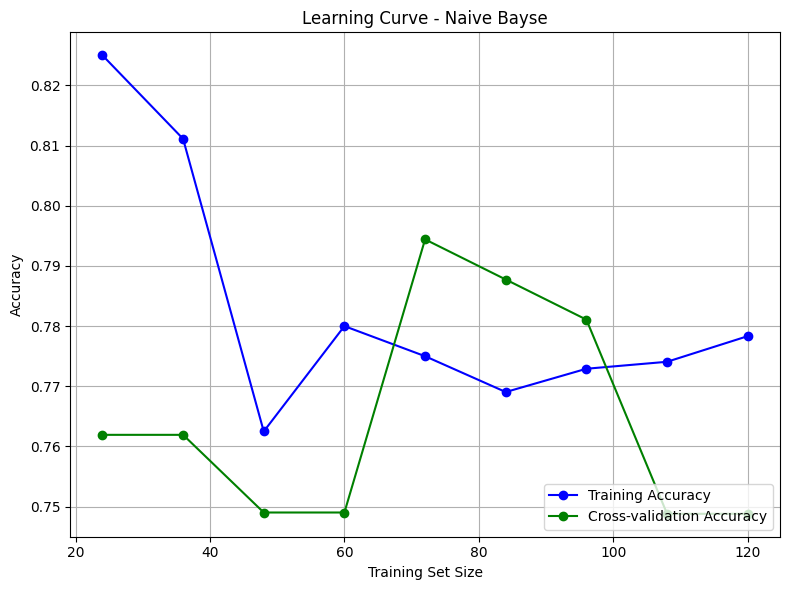


**1.4.6 Loss Curve**


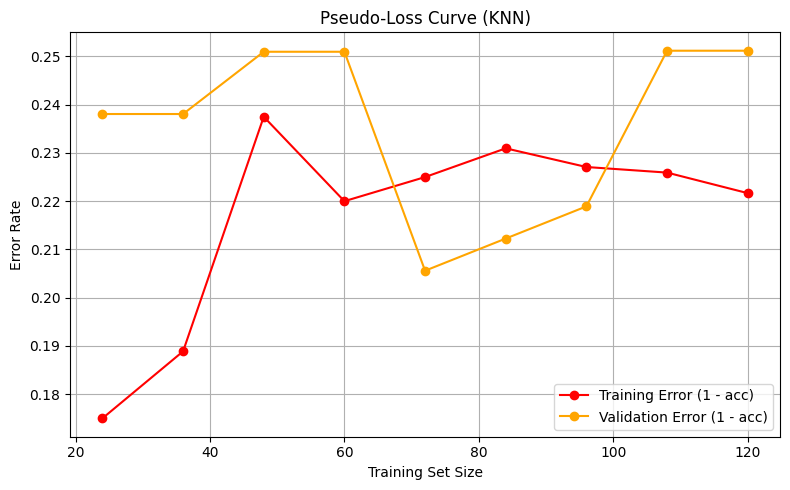


**Importance of Balanced Metrics**

While accuracy is a common metric, it alone can be misleading in imbalanced datasets. Therefore, recall, precision, and F1-score were used to provide a more balanced assessment. For instance,

The recall value indicates the model's ability to correctly identify positive cases (especially important in domains such as medical diagnostics).

The F1-score offers a trade-off between precision and recall, making it suitable for imbalanced classification tasks.

The AUC score for the best model was Random Forest model which suggests good discriminative ability between the positive and negative classes.

**Model Selection Justification**

The final model was selected not only based on its performance on the training and validation data, but most importantly on its consistent performance on the test set. The chosen model balanced predictive power and robustness, showing no significant drop in test performance, which is essential for real-world deployment.
